# Comparative host-coronavirus protein interaction networks reveal pan-viral disease mechanisms

**DOI:** 10.1126/science.abe9403

**Published:** 2020-10-15

**Authors:** David E. Gordon, Joseph Hiatt, Mehdi Bouhaddou, Veronica V. Rezelj, Svenja Ulferts, Hannes Braberg, Alexander S. Jureka, Kirsten Obernier, Jeffrey Z. Guo, Jyoti Batra, Robyn M. Kaake, Andrew R. Weckstein, Tristan W. Owens, Meghna Gupta, Sergei Pourmal, Erron W. Titus, Merve Cakir, Margaret Soucheray, Michael McGregor, Zeynep Cakir, Gwendolyn Jang, Matthew J. O’Meara, Tia A. Tummino, Ziyang Zhang, Helene Foussard, Ajda Rojc, Yuan Zhou, Dmitry Kuchenov, Ruth Hüttenhain, Jiewei Xu, Manon Eckhardt, Danielle L. Swaney, Jacqueline M. Fabius, Manisha Ummadi, Beril Tutuncuoglu, Ujjwal Rathore, Maya Modak, Paige Haas, Kelsey M. Haas, Zun Zar Chi Naing, Ernst H. Pulido, Ying Shi, Inigo Barrio-Hernandez, Danish Memon, Eirini Petsalaki, Alistair Dunham, Miguel Correa Marrero, David Burke, Cassandra Koh, Thomas Vallet, Jesus A. Silvas, Caleigh M. Azumaya, Christian Billesbølle, Axel F. Brilot, Melody G. Campbell, Amy Diallo, Miles Sasha Dickinson, Devan Diwanji, Nadia Herrera, Nick Hoppe, Huong T. Kratochvil, Yanxin Liu, Gregory E. Merz, Michelle Moritz, Henry C. Nguyen, Carlos Nowotny, Cristina Puchades, Alexandrea N. Rizo, Ursula Schulze-Gahmen, Amber M. Smith, Ming Sun, Iris D. Young, Jianhua Zhao, Daniel Asarnow, Justin Biel, Alisa Bowen, Julian R. Braxton, Jen Chen, Cynthia M. Chio, Un Seng Chio, Ishan Deshpande, Loan Doan, Bryan Faust, Sebastian Flores, Mingliang Jin, Kate Kim, Victor L. Lam, Fei Li, Junrui Li, Yen-Li Li, Yang Li, Xi Liu, Megan Lo, Kyle E. Lopez, Arthur A. Melo, Frank R. Moss, Phuong Nguyen, Joana Paulino, Komal Ishwar Pawar, Jessica K. Peters, Thomas H. Pospiech, Maliheh Safari, Smriti Sangwan, Kaitlin Schaefer, Paul V. Thomas, Aye C. Thwin, Raphael Trenker, Eric Tse, Tsz Kin Martin Tsui, Feng Wang, Natalie Whitis, Zanlin Yu, Kaihua Zhang, Yang Zhang, Fengbo Zhou, Daniel Saltzberg, QCRG Structural Biology Consortium, Anthony J. Hodder, Amber S. Shun-Shion, Daniel M. Williams, Kris M. White, Romel Rosales, Thomas Kehrer, Lisa Miorin, Elena Moreno, Arvind H. Patel, Suzannah Rihn, Mir M. Khalid, Albert Vallejo-Gracia, Parinaz Fozouni, Camille R. Simoneau, Theodore L. Roth, David Wu, Mohd Anisul Karim, Maya Ghoussaini, Ian Dunham, Francesco Berardi, Sebastian Weigang, Maxime Chazal, Jisoo Park, James Logue, Marisa McGrath, Stuart Weston, Robert Haupt, C. James Hastie, Matthew Elliott, Fiona Brown, Kerry A. Burness, Elaine Reid, Mark Dorward, Clare Johnson, Stuart G. Wilkinson, Anna Geyer, Daniel M. Giesel, Carla Baillie, Samantha Raggett, Hannah Leech, Rachel Toth, Nicola Goodman, Kathleen C. Keough, Abigail L. Lind, Zoonomia Consortium, Reyna J. Klesh, Kafi R. Hemphill, Jared Carlson-Stevermer, Jennifer Oki, Kevin Holden, Travis Maures, Katherine S. Pollard, Andrej Sali, David A. Agard, Yifan Cheng, James S. Fraser, Adam Frost, Natalia Jura, Tanja Kortemme, Aashish Manglik, Daniel R. Southworth, Robert M. Stroud, Dario R. Alessi, Paul Davies, Matthew B. Frieman, Trey Ideker, Carmen Abate, Nolwenn Jouvenet, Georg Kochs, Brian Shoichet, Melanie Ott, Massimo Palmarini, Kevan M. Shokat, Adolfo García-Sastre, Jeremy A. Rassen, Robert Grosse, Oren S. Rosenberg, Kliment A. Verba, Christopher F. Basler, Marco Vignuzzi, Andrew A. Peden, Pedro Beltrao, Nevan J. Krogan

**Affiliations:** 1 Quantitative Biosciences Institute (QBI) COVID-19 Research Group (QCRG), San Francisco, CA 94158, USA.; 2 QBI, University of California, San Francisco, CA 94158, USA.; 3 Department of Cellular and Molecular Pharmacology, University of California, San Francisco, CA 94158, USA.; 4 J. David Gladstone Institutes, San Francisco, CA 94158, USA.; 5 Medical Scientist Training Program, University of California, San Francisco, CA 94143, USA.; 6 Department of Microbiology and Immunology, University of California, San Francisco, CA 94143, USA.; 7 Biomedical Sciences Graduate Program, University of California, San Francisco, CA 94143, USA.; 8 Viral Populations and Pathogenesis Unit, CNRS UMR 3569, Institut Pasteur, 75724, Paris, cedex 15, France.; 9 Institute for Clinical and Experimental Pharmacology and Toxicology I, University of Freiburg, 79104 Freiburg, Germany.; 10 Center for Microbial Pathogenesis, Institute for Biomedical Sciences, Georgia State University, Atlanta, GA 30303, USA.; 11 Aetion, Inc., New York, NY 10001, USA.; 12 QBI Coronavirus Research Group Structural Biology Consortium, University of California, San Francisco, CA 94158, USA.; 13 Department of Computational Medicine and Bioinformatics, University of Michigan, Ann Arbor, MI 48109, USA.; 14 Department of Pharmaceutical Chemistry, University of California, San Francisco, CA 94158, USA.; 15 Howard Hughes Medical Institute, San Francisco, CA 94158, USA.; 16 European Molecular Biology Laboratory, European Bioinformatics Institute (EMBL-EBI), Wellcome Genome Campus, Hinxton, Cambridgeshire CB10 1SD, UK.; 17 Division of Basic Sciences, Fred Hutchinson Cancer Research Center, Seattle, WA 98109, USA.; 18 Beam Therapeutics, Cambridge, MA 02139, USA.; 19 Department of Bioengineering and Therapeutic Sciences, University of California, San Francisco, CA 94158, USA.; 20 Department of Biomedical Science, Centre for Membrane Interactions and Dynamics, University of Sheffield, Firth Court, Sheffield S10 2TN, UK.; 21 Department of Microbiology, Icahn School of Medicine at Mount Sinai, New York, NY 10029, USA.; 22 Global Health and Emerging Pathogens Institute, Icahn School of Medicine at Mount Sinai, New York, NY 10029, USA.; 23 MRC–University of Glasgow Centre for Virus Research, Glasgow G61 1QH, Scotland, UK.; 24 Wellcome Trust Sanger Institute, Wellcome Genome Campus, Hinxton, Cambridgeshire CB10 1SA, UK.; 25 Open Targets, Wellcome Genome Campus, Hinxton, Cambridgeshire CB10 1SD, UK.; 26 Dipartimento di Farmacia-Scienze del Farmaco, Università degli Studi di Bari ‘ALDO MORO’, Via Orabona, 4 70125, Bari, Italy.; 27 Institute of Virology, Medical Center–University of Freiburg, 79104 Freiburg, Germany.; 28 Département de Virologie, CNRS UMR 3569, Institut Pasteur, Paris 75015, France.; 29 Department of Medicine, University of California, San Diego, CA 92093, USA.; 30 Department of Microbiology and Immunology, University of Maryland School of Medicine, Baltimore, MD 21201, USA.; 31 MRC Protein Phosphorylation and Ubiquitylation Unit, College of Life Sciences, University of Dundee, Dundee DD1 5EH, UK.; 32 HealthVerity, Philadelphia, PA 19103, USA.; 33 Department of Neurology, University of California, San Francisco, CA 94143, USA.; 34 Synthego Corporation, Redwood City, CA 94063, USA.; 35 Department of Epidemiology & Biostatistics, University of California, San Francisco, CA 94158, USA.; 36 Chan-Zuckerberg Biohub, San Francisco, CA 94158, USA.; 37 Department of Biochemistry and Biophysics, University of California, San Francisco, CA 94158, USA.; 38 Cardiovascular Research Institute, University of California, San Francisco, CA 94158, USA.; 39 The University of California, Berkeley–University of California, San Francisco Graduate Program in Bioengineering, University of California, San Francisco, CA 94158, USA.; 40 Department to Bioengineering, University of California, San Diego, CA 92093, USA.; 41 Department of Medicine, University of California, San Francisco, CA 94143, USA.; 42 Department of Medicine, Division of Infectious Diseases, Icahn School of Medicine at Mount Sinai, New York, NY 10029, USA.; 43 The Tisch Cancer Institute, Icahn School of Medicine at Mount Sinai, New York, NY 10029, USA.; 44 Centre for Integrative Biological Signaling Studies (CIBSS), University of Freiburg, 79104 Freiburg, Germany.

## Abstract

The COVID-19 pandemic, caused by severe acute respiratory syndrome coronavirus 2 (SARS-CoV-2), is a grave threat to public health and the global economy. SARS-CoV-2 is closely related to the more lethal but less transmissible coronaviruses SARS-CoV-1 and Middle East respiratory syndrome coronavirus (MERS-CoV). Here, we have carried out comparative viral-human protein-protein interaction and viral protein localization analyses for all three viruses. Subsequent functional genetic screening identified host factors that functionally impinge on coronavirus proliferation, including Tom70, a mitochondrial chaperone protein that interacts with both SARS-CoV-1 and SARS-CoV-2 ORF9b, an interaction we structurally characterized using cryo–electron microscopy. Combining genetically validated host factors with both COVID-19 patient genetic data and medical billing records identified molecular mechanisms and potential drug treatments that merit further molecular and clinical study.

## How lethal coronaviruses engage hosts

Severe acute respiratory syndrome coronavirus 2 (SARS-CoV-2) is closely related to the deadly coronaviruses SARS-CoV-1 and Middle East respiratory syndrome coronavirus (MERS-CoV). Considerable efforts are focused on developing treatments, and therapies that work across coronaviruses would be particularly valuable. Shedding light on the host factors hijacked by the viruses, Gordon *et al.* mapped the interactions between viral and human proteins for SARS-CoV-2, SARS-CoV-1, and MERS-CoV; analyzed the localization of viral proteins in human cells; and used genetic screening to identify host factors that either enhance or inhibit viral infection. For a subset of the interactions essential for the virus life cycle, the authors determined the cryo–electron microscopy structures and mined patient data to understand how targeting host factors may be relevant to clinical outcomes.

*Science*, this issue p. eabe9403

## Abstract (structured)

**INTRODUCTION:**

The emergence of three lethal coronaviruses in <20 years and the urgency of the COVID-19 pandemic have prompted efforts to develop new therapeutic strategies, including by repurposing existing agents. After performing a comparative analysis of the three pathogenic human coronaviruses severe acute respiratory syndrome coronavirus 1 (SARS-CoV-1), SARS-CoV-2, and Middle East respiratory syndrome coronavirus (MERS-CoV), we identified shared biology and host-directed drug targets to prioritize therapeutics with potential for rapid deployment against current and future coronavirus outbreaks.

**RATIONALE:**

Expanding on our recent SARS-CoV-2 interactome, we mapped the virus-host protein-protein interactions for SARS-CoV-1 and MERS-CoV and assessed the cellular localization of each viral protein across the three strains. We conducted two genetic screens of SARS-CoV-2 interactors to prioritize functionally-relevant host factors and structurally characterized one virus-host interaction. We then tested the clinical relevance of three more host factors by assessing risk in genetic cohorts or observing effectiveness of host factor–targeting drugs in real-world evidence.

**RESULTS:**

Quantitative comparison of the 389 interactors of SARS-CoV-2, 366 of SARS-CoV-1, and 296 of MERS-CoV highlighted interactions with host processes that are conserved across all three viruses, including where nonorthologous proteins from different virus strains seem to fill similar roles. We also localized each individually-expressed viral protein by microscopy and then raised and validated antisera against 14 SARS-CoV-2 proteins to determine their localization during infection.

On the basis of two independent genetic perturbation screens, we identified 73 host factors that, when depleted, caused significant changes in SARS-CoV-2 replication. From this list of potential drug targets, we validated the biological and clinical relevance of Tom70, IL17RA, PGES-2, and SigmaR1.

A 3-Å cryo–electron microscopy structure of Tom70, a mitochondrial import receptor, in complex with SARS-CoV-2 ORF9b, provides insight into how ORF9b may modulate the host immune response. Using curated genome-wide association study data, we found that individuals with genotypes corresponding to higher soluble IL17RA levels in plasma are at decreased risk of COVID-19 hospitalization.

To demonstrate the value of our data for drug repurposing, we identified SARS-CoV-2 patients who were prescribed drugs against prioritized targets and asked how they fared compared with carefully matched patients treated with clinically similar drugs that do not inhibit SARS-CoV-2. Both indomethacin, an inhibitor of host factor PGES-2, and typical antipsychotics, selected for their interaction with sigma receptors, showed effectiveness against COVID-19 compared with celecoxib and atypical antipsychotics, respectively.

**CONCLUSION:**

By employing an integrative and collaborative approach, we identified conserved mechanisms across three pathogenic coronavirus strains and further investigated potential drug targets. This versatile approach is broadly applicable to other infectious agents and disease areas.

## Teaser

Comparison of host interactions of three lethal coronaviruses identifies commonly hijacked pathways and potential drug targets.

## Graphical abstract

**Figure:**
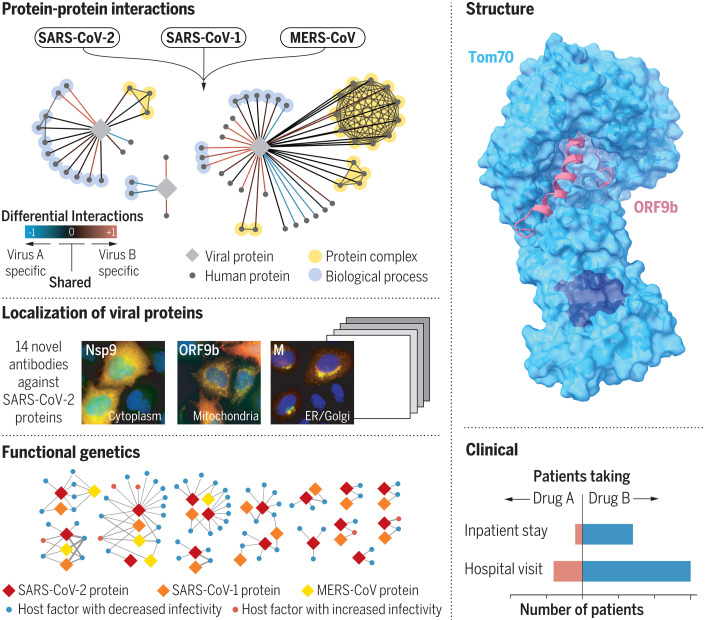
Overview of the approaches taken for systemic and functional comparison of pathogenic human coronaviruses. (Left) Viral-human protein-protein interaction network mapping, viral protein localization studies, and functional genetic screens provide key insights into the shared and individual characteristics of each virus. (Right) Structural studies and hypothesis testing in clinical datasets demonstrate the utility of this approach for prioritizing therapeutic strategies. Nsp, nonstructural protein; ORF, open reading frame; ER, endoplasmic reticulum.

In the past two decades, three deadly human respiratory syndromes associated with coronavirus (CoV) infections have emerged: severe acute respiratory syndrome (SARS) in 2002, Middle East respiratory syndrome (MERS) in 2012, and COVID-19 in 2019. These three diseases are caused by the zoonotic coronaviruses severe acute respiratory syndrome coronavirus 1 (SARS-CoV-1), Middle East respiratory syndrome coronavirus (MERS-CoV), and SARS-CoV-2 (*1*), respectively. Before their emergence, human coronaviruses were associated with usually mild respiratory illness. To date, SARS-CoV-2 has sickened millions and killed more than 1 million people worldwide. This unprecedented challenge has prompted widespread efforts to develop vaccine and antiviral strategies, including repurposed therapeutics, which offer the potential for treatments with known safety profiles and short development timelines. The successful repurposing of the antiviral nucleoside analog Remdesivir (*2*) as well as the host-directed anti-inflammatory steroid dexamethasone (*3*) provide clear proof that existing compounds can be crucial tools in the fight against COVID-19. Despite these promising examples, there is still no curative treatment for COVID-19. Additionally, as with any virus, the search for effective antiviral strategies could be complicated over time by the continued evolution of SARS-CoV-2 and possible resulting drug resistance (*4*).

Current endeavors are appropriately focused on SARS-CoV-2 because of the severity and urgency of the ongoing pandemic. However, the frequency with which highly virulent coronavirus strains have emerged highlights an additional need to identify promising targets for broad coronavirus inhibitors with high barriers to resistance mutations and the potential for rapid deployment against future emerging strains. Although traditional antivirals target viral enzymes that are often subject to mutation and thus the development of drug resistance, targeting the host proteins required for viral replication is a strategy that can avoid resistance and lead to therapeutics with the potential for broad-spectrum activity because families of viruses often exploit common cellular pathways and processes.

Here, we identified shared biology and potential drug targets among the three highly pathogenic human coronavirus strains. We expanded on our recently published map of virus-host protein interactions for SARS-CoV-2 (*5*) and mapped the full interactomes of SARS-CoV-1 and MERS-CoV. We investigated the localization of viral proteins across strains and quantitatively compared the virus-human interactions for each virus. Using functional genetics and structural analysis of selected host-dependency factors, we identified drug targets and performed real-world analysis on clinical data from COVID-19 patients.

## A cross-coronavirus study of protein function

**Fig. 1:**
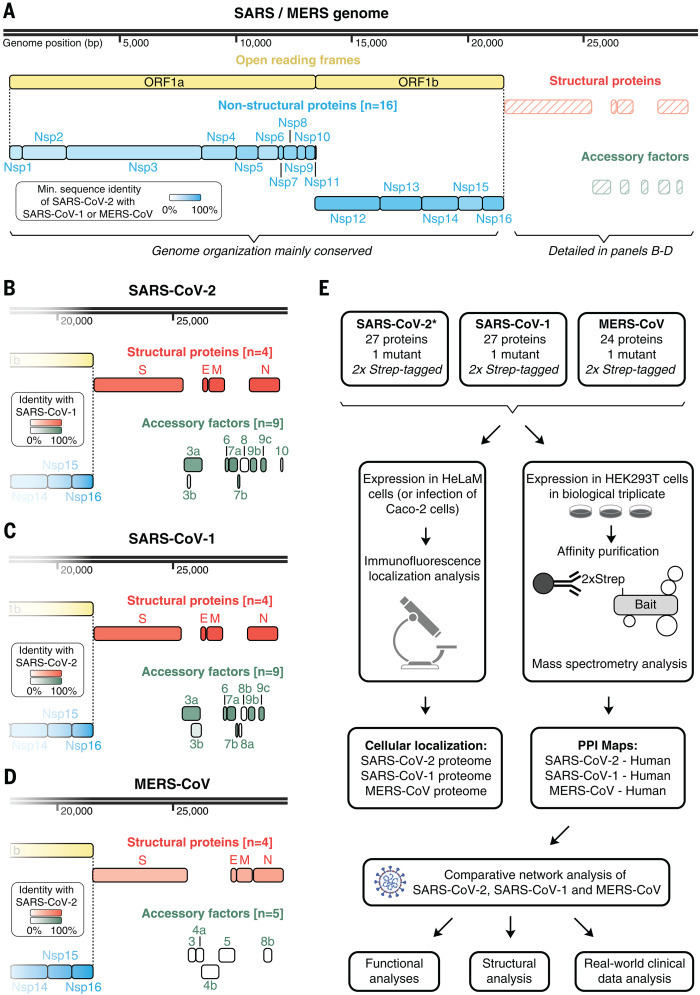
Coronavirus genome annotations and integrative analysis overview. (**A**) Genome annotation of SARS-CoV-2, SARS-CoV-1, and MERS-CoV with putative protein coding genes highlighted. Intensity of filled color indicates the lowest sequence identity between SARS-CoV-2 and SARS-CoV-1 or between SARS-CoV-2 and MERS. (**B** to **D**) Genome annotation of structural protein genes for SARS-CoV-2 (B), SARS-CoV-1 (C), and MERS-CoV (D). Color intensity indicates sequence identity to specified virus. (**E**) Overview of comparative coronavirus analysis. Proteins from SARS-CoV-2, SARS-CoV-1, and MERS-CoV were analyzed for their protein interactions and subcellular localization, and these data were integrated for comparative host interaction network analysis, followed by functional, structural, and clinical data analyses for exemplary virus-specific and pan-viral interactions. The asterisk indicates that the SARS-CoV-2 interactome was previously published in a separate study (*5*). SARS, both SARS-CoV-1 and SARS-CoV-2; MERS, MERS-CoV; Nsp, nonstructural protein; ORF, open reading frame.

A central goal of this study is to understand, from a systems level, the conservation of target proteins and cellular processes between SARS-CoV-2, SARS-CoV-1, and MERS-CoV, thereby identifying shared vulnerabilities that can be targeted with antiviral therapeutics. All three strains encode four homologous structural proteins (E, M, N, and S) and 16 nonstructural proteins (Nsps). The latter are proteolytically cleaved from a polyprotein precursor that is expressed from one large open reading frame (ORF), ORF1ab (Fig. 1A). Additionally, coronaviruses contain a variable number of accessory factors encoded by ORFs. Although the genome organization and sequence of ORF1ab is mainly conserved between the three viruses under study, it diverges markedly in the region encoding the accessory factors, especially between MERS-CoV and the two SARS coronaviruses (Fig. 1, A to D, and table S1). These differences in conservation of genes and genome organization are linked to differences in host-targeting systems that we have studied through large-scale protein localization and interaction profiling (Fig. 1E). Building on our earlier work on the interactome of SARS-CoV-2 (*5*), we identified the host factors physically interacting with each SARS-CoV-1 and MERS-CoV viral protein. To this end, structural proteins, mature Nsps, and predicted ORF proteins were codon optimized, 2xStrep tagged, and cloned into a mammalian expression vector (figs. S1 and S2; see below and Materials and methods section). Each protein construct was transfected into HEK293T cells and affinity purified, and high-confidence interactors were identified by mass spectrometry (MS) and scored using SAINTexpress (significance analysis of interactome) and MiST (mass spectrometry interaction statistics) scoring algorithms (*6*, *7*) (table S2 and figs. S3 to S6). Additionally, we performed MS analysis on SARS-CoV-2 Nsp16, which was not analyzed in our earlier work (*5*) (table S2 and fig. S7). In all, we now report 389 high-confidence interactors for SARS-CoV-2, 366 interactors for SARS-CoV-1, and 296 interactors for MERS-CoV (table S2).

## Conserved coronavirus proteins often retain the same cellular localization

**Fig. 2:**
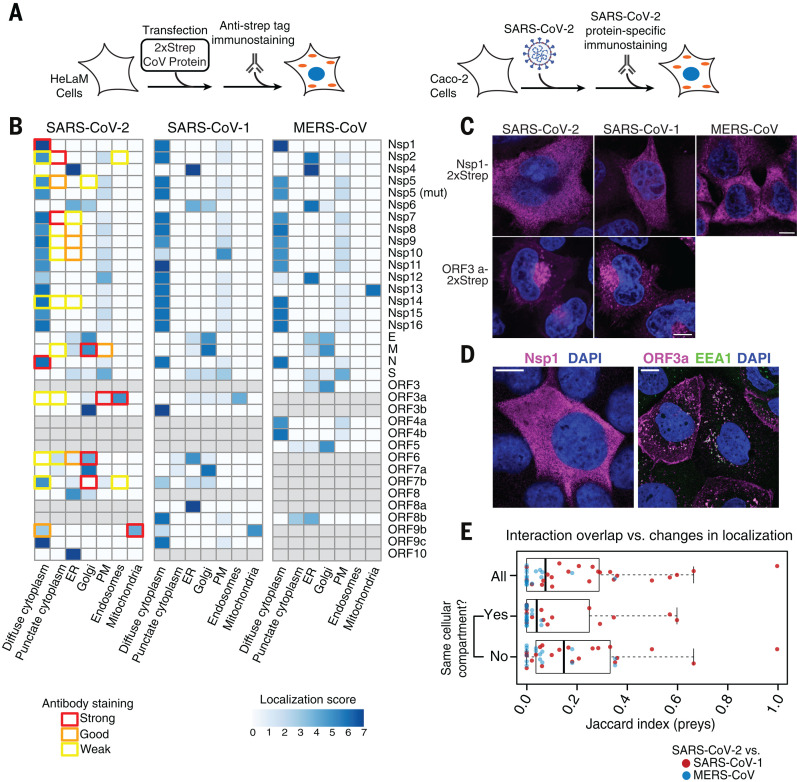
Coronavirus protein localization analysis. (**A**) Overview of experimental design to determine localization of Strep-tagged SARS-CoV-2, SARS-CoV-1, and MERS-CoV proteins in HeLaM cells (left) or of viral proteins upon SARS-CoV-2 infection in Caco-2 cells (right). (**B**) Relative localization for all coronavirus proteins across viruses expressed individually (blue color bar) or in SARS-CoV-2–infected cells (colored box outlines). (**C** and **D**) Localization of Nsp1 and ORF3a expressed individually (C) or during infection (D); for representative images of all tagged constructs and viral proteins imaged during infection, see figs. S8 to S14 and fig. S15, respectively. Scale bars, 10 μm. (**E**) Prey overlap per bait measured as Jaccard index comparing SARS-CoV-2 versus SARS-CoV-1 (red dots) and SARS-CoV-2 versus MERS-CoV (blue dots) for all viral baits (all), viral baits found in the same cellular compartment (yes), and viral baits found in different compartments (no).

As protein localization can provide important information regarding function, we assessed the cellular localization of individually expressed coronavirus proteins in addition to mapping their interactions (Fig. 2A and Materials and methods). Immunofluorescence localization analysis of all 2xStrep-tagged SARS-CoV-2, SARS-CoV-1, and MERS-CoV proteins highlights similar patterns of localization for most shared protein homologs in HeLaM cells (Fig. 2B), which supports the hypothesis that conserved proteins share functional similarities. A notable exception is Nsp13, which appears to localize to the cytoplasm for SARS-CoV-2 and SARS-CoV-1, but to the mitochondria for MERS-CoV (Fig. 2B, figs. S8 to S13, and table S3). To assess the localization of SARS-CoV-2 proteins in the context of infected cells, we raised antibodies against 20 SARS-CoV-2 proteins and validated them with the individually expressed 2xStrep-tagged proteins (fig. S14). Using the 14 antibodies with confirmed specificity, we observed that localization of viral proteins in infected Caco-2 cells sometimes differed from their localization when expressed individually (Fig. 2B, fig. S15, and table S3). This likely results from recruitment of viral proteins and complexes into replication compartments, as well as from remodeling of the secretory pathway during viral infection. Such differences could also result from mislocalization caused by protein tagging. For example, the localization of expressed ORF7B does not match the known SARS-CoV-1 Golgi localization seen in the infection state. For proteins such as Nsp1 and ORF3a, which are not known to be involved in viral replication, their localization is consistent both when expressed individually and in the context of viral infection (Fig. 2, C and D). We have compared the localization of the expressed viral proteins with the localization of their interaction partners using a cellular compartment gene ontology (GO) enrichment analysis (fig. S16). Several examples exist where the localization of the viral protein is in agreement with the localization of the interaction partners, including enrichment of the nuclear pore for Nsp9 interactors and endoplasmic reticulum (ER) enrichment for interactions with ORF8.

Our localization studies suggest that most orthologous proteins have the same localization across the viruses (Fig. 2B). Moreover, small changes in localization, as observed for some viral proteins across strains, do not coincide with strong changes in virus-host protein interactions (Fig. 2E). Overall, these results suggest that changes in protein localization, as measured by expressed, tagged proteins, are not common and therefore are unlikely to be a major source of differences in host-targeting mechanisms.

## Comparison of host-targeted processes identifies conserved mechanisms with divergent implementations

**Fig. 3:**
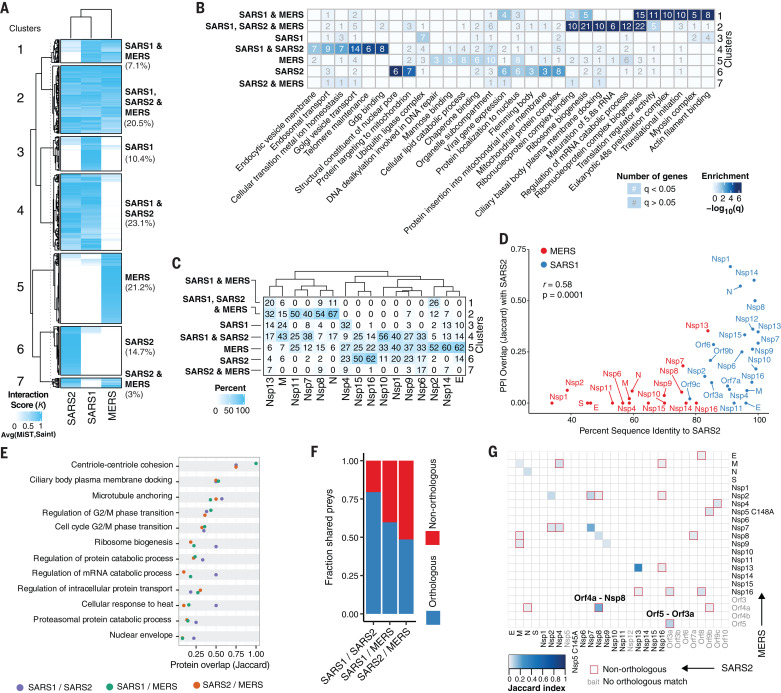
Comparative analysis of coronavirus-host interactomes. (**A**) Clustering analysis (*K*-means) of interactors from SARS-CoV-2, SARS-CoV-1, and MERS-CoV, weighted according to the average between their MiST and SAINT scores (interaction score *K*). Included are only viral protein baits represented amongst all three viruses and interactions that pass the high-confidence scoring threshold for at least one virus. Seven clusters highlight all possible scenarios of shared versus individual interactions, and percentages of total interactions are noted. (**B**) GO enrichment analysis of each cluster from (A), with the top six most-significant terms per cluster. Color indicates −log_10_(*q*), and the number of genes with significant (*q* < 0.05; white) or nonsignificant enrichment (*q* > 0.05; gray) is shown. (**C**) Percentage of interactions for each viral protein belonging to each cluster identified in (A). (**D**) Correlation between protein sequence identity and PPI overlap (Jaccard index) comparing SARS-CoV-2 and SARS-CoV-1 (blue) or MERS-CoV (red). Interactions for PPI overlap are derived from the final thresholded list of interactions per virus. (**E**) GO biological process terms significantly enriched (*q* < 0.05) for all three virus PPIs with Jaccard index indicating overlap of genes from each term for pairwise comparisons between SARS-CoV-1 and SARS-CoV-2 (purple), SARS-CoV-1 and MERS-CoV (green), and SARS-CoV-2 and MERS-CoV (orange). (**F**) Fraction of shared preys between orthologous (blue) and nonorthologous (red) viral protein baits. (**G**) Heatmap depicting overlap in PPIs (Jaccard index) between each bait from SARS-CoV-2 and MERS-CoV. Baits in gray were not assessed, do not exist, or do not have high-confidence interactors in the compared virus. Nonorthologous bait interactions are highlighted with a red square. GO, gene ontology; PPI, protein-protein interaction; SARS2, SARS-CoV-2; SARS1, SARS-CoV-1; MERS, MERS-CoV.

To study the conservation of targeted host factors and processes, we first used a clustering approach (Materials and methods) to compare the overlap in protein interactions for the three viruses (Fig. 3A). We defined seven clusters of virus-host interactions corresponding to those that are specific to each virus or are shared among sets of viruses. The largest pairwise overlap was observed between SARS-CoV-1 and SARS-CoV-2 (Fig. 3A), as is expected from their closer evolutionary relationship. A functional enrichment analysis (Fig. 3B and table S4) highlighted host processes that are targeted through interactions conserved across all three viruses, including ribosome biogenesis and regulation of RNA metabolism. Conserved interactions between SARS-CoV-1 and SARS-CoV-2—but not MERS-CoV—were enriched in endosomal and Golgi vesicle transport (Fig. 3B). Despite the small fraction (7.1%) of interactions conserved between SARS-CoV-1 and MERS-CoV—but not SARS-CoV-2—these were strongly enriched in translation initiation and myosin complex proteins (Fig. 3B).

We next asked whether the conserved interactions were specific for certain viral proteins (Fig. 3C) and found that some proteins (M, N, Nsp7, Nsp8, and Nsp13) showed a disproportionately high fraction of shared interactions conserved across the three viruses. This suggests that the processes targeted by these proteins may be more essential and more likely to be required for other emerging coronaviruses. Such differences in conservation of interactions should be encoded, to some extent, in the degree of sequence differences. Comparing pairs of homologous proteins shared between SARS-CoV-2 and SARS-CoV-1 or MERS-CoV, we observed a significant correlation between sequence conservation and protein-protein interaction (PPI) similarity (calculated as Jaccard index) [Fig. 3D; correlation coefficient (*r*) = 0.58, *P* = 0.0001]. This shows that the evolution of protein sequences strongly determines the divergence in virus-host interactions.

While studying the function of host proteins interacting with each virus, we noted that some shared cellular processes were targeted by different interactions across the viruses. To study this in more detail, we identified the cellular processes significantly enriched in the interactomes of all three viruses (fig. S17A and table S4) and ranked them by the degree of overlapping proteins (Fig. 3E). This identified proteins related to the nuclear envelope, proteasomal catabolism, cellular response to heat, and regulation of intracellular protein transport as biological functions that are hijacked by these viruses through different human proteins. Additionally, we found that up to 51% of protein interactions with a conserved human target occurred via a different (nonorthologous) viral protein (Fig. 3F), and, in some cases, the overlap of interactions for two nonorthologous virus baits was greater than that for the orthologous pair (Fig. 3G and fig. S17, B and C). For example, several interacting proteins of SARS-CoV-2 Nsp8 are also targeted by MERS-CoV ORF4a, and interactions of MERS-CoV ORF5 share interactors with SARS-CoV-2 ORF3a (Fig. 3G). In the case of Nsp8, we found some degree of structural homology between its C-terminal region and a predicted structural model of ORF4a (Materials and methods and fig. S17D), which is indicative of a possible common interaction mechanism.

We find that sequence differences determine the degree of changes in virus-host interactions and that often the same cellular process can be targeted by different viral or host proteins. These results suggest a degree of plasticity in the way that these viruses can control a given biological process in the host cell.

## Quantitative differential interaction scoring identifies interactions conserved between coronaviruses

**Fig. 4:**
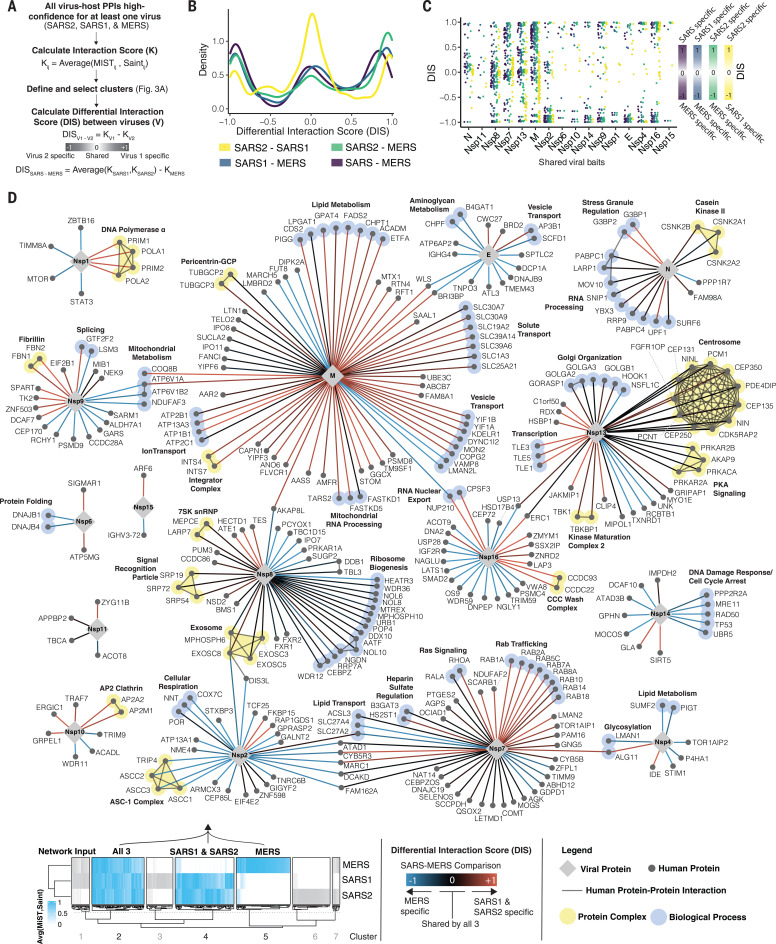
Comparative differential interaction analysis reveals shared virus-host interactions. (**A**) Flowchart depicting calculation of DIS values using the average between the SAINT and MiST scores between every bait (*i*) and prey (*j*) to derive interaction score (*K*). The DIS is the difference between the interaction scores from each virus. The modified DIS (SARS-MERS) compares the average *K* from SARS-CoV-1 and SARS-CoV-2 to that of MERS-CoV (see Materials and methods). Only viral bait proteins shared between all three viruses are included. (**B**) Density histogram of the DISs for all comparisons. (**C**) Dot plot depicting the DISs of interactions from viral bait proteins shared between all three viruses, ordered left to right by the mean DIS per viral bait. (**D**) Virus-human PPI map depicting the SARS-MERS comparison [purple in (B) and (C)]. The network depicts interactions derived from cluster 2 (all three viruses), cluster 4 (SARS-CoV-1 and SARS-CoV-2), and cluster 5 (MERS-CoV only). Edge color denotes DIS: red indicates interactions specific to SARS-CoV-1 and SARS-CoV-2 but absent in MERS-CoV; blue indicates interactions specific to MERS-CoV but absent from both SARS-CoV-1 and SARS-CoV-2; and black indicates interactions shared between all three viruses. Human-human interactions (thin dark gray line) and proteins sharing the same protein complexes or biological processes (light yellow or light blue highlighting, respectively) are shown. Host-host physical interactions, protein complex definitions, and biological process groupings are derived from CORUM (*46*), GO (biological process), and manually curated from literature sources. Thin dashed gray lines are used to indicate the placement of node labels when adjacent node labels would have otherwise been obscured. DIS, differential interaction score; SARS2, SARS-CoV-2; SARS1, SARS-CoV-1; MERS, MERS-CoV; SARS, both SARS-CoV-1 and SARS-CoV-2.

The identification of virus-host interactions conserved across pathogenic coronaviruses provides the opportunity to reveal host targets that may remain essential for these and other emerging coronaviruses. For a quantitative comparison of each virus-human interaction from viral baits shared by all three viruses, we developed a differential interaction score (DIS). A DIS is calculated between any pair of viruses and is defined as the difference between the interaction scores (*K*) from each virus (Fig. 4A, table S5, and Materials and methods). This kind of comparative analysis is beneficial as it permits the recovery of conserved interactions that may fall just below strict cutoffs. For each comparison, a DIS was calculated for interactions residing in certain clusters as defined in the previous analysis (see Fig. 3A). For example, for the SARS-CoV-2 to MERS-CoV comparison, a DIS was computed for interactions residing in all clusters except cluster 3, where interactions are either not found or scores were very low for both SARS-CoV-2 and MERS-CoV. A DIS of 0 indicates that the interaction is confidently shared between the two viruses being compared, whereas a DIS of +1 or −1 indicates that the host-protein interaction is specific for the virus listed first or second, respectively.

In agreement with our previous results (Fig. 3A), DIS values for the comparison between SARS-CoV-2 and SARS-CoV-1 are enriched near zero, which indicates a high number of shared interactions (Fig. 4B, yellow). By contrast, comparing interactions from either SARS-CoV-1 or SARS-CoV-2 with MERS-CoV resulted in DIS values closer to ±1, which indicates a higher divergence (Fig. 4B, blue and green). The breakdown of DISs by homologous viral proteins reveals a high similarity of interactions for proteins N, Nsp8, Nsp7, and Nsp13 (Fig. 4C), reinforcing the observations made by overlapping thresholded interactions (Fig. 3, C and D). As the greatest dissimilarity was observed between the SARS coronaviruses and MERS-CoV, we computed a fourth DIS (SARS-MERS) by averaging *K* from SARS-CoV-1 and SARS-CoV-2 before calculating the difference with MERS-CoV (Fig. 4, B and C, purple). We next created a network visualization of the SARS-MERS comparison (Fig. 4D), permitting an appreciation of SARS-specific (red; DIS near +1) versus MERS-specific (blue; DIS near −1) interactions as well as those conserved between all three coronavirus species (black; DIS near 0). SARS-specific interactions include DNA polymerase α interacting with Nsp1, stress granule regulators interacting with N protein, TLE transcription factors interacting with Nsp13, and AP2 clathrin interacting with Nsp10. Notable MERS-CoV–specific interactions include mammalian target of rapamycin (mTOR) and Stat3 interacting with Nsp1; DNA damage response components p53 (*TP53*), MRE11, RAD50, and UBR5 interacting with Nsp14; and the activating signal cointegrator 1 (ASC-1) complex interacting with Nsp2. Interactions shared between all three coronaviruses include casein kinase II and RNA processing regulators interacting with N protein; inosine 5′-monophosphate (IMP) dehydrogenase 2 (*IMPDH2*) interacting with Nsp14; centrosome, protein kinase A, and TBK1 interacting with Nsp13; and the signal recognition particle, 7SK small nuclear ribonucleoprotein (snRNP), exosome, and ribosome biogenesis components interacting with Nsp8 (Fig. 4D).

## Cell-based genetic screens identify SARS-CoV-2 host-dependency factors

**Fig. 5:**
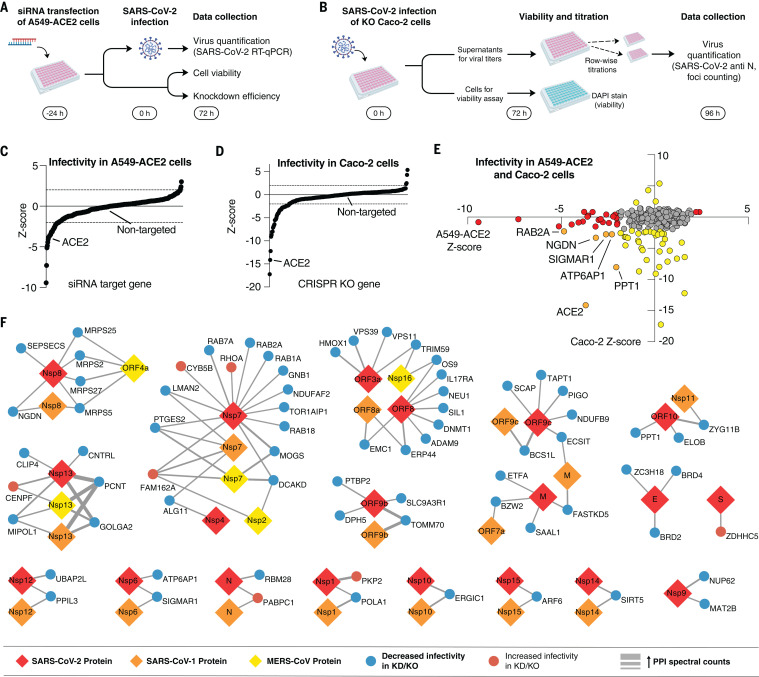
Functional interrogation of SARS-CoV-2 interactors using genetic perturbations. (**A**) A549-ACE2 cells were transfected with siRNA pools targeting each of the human genes from the SARS-CoV-2 interactome, followed by infection with SARS-CoV-2 and virus quantification using RT-qPCR. Cell viability and knockdown efficiency in uninfected cells was determined in parallel. (**B**) Caco-2 cells with CRISPR knockouts (KO) of each human gene from the SARS-CoV-2 interactome were infected with SARS-CoV-2, and supernatants were serially diluted and plated onto Vero E6 cells for quantification. Viabilities of the uninfected CRISPR knockout cells after infection were determined in parallel by DAPI staining. (**C** and **D**) Plot of results from the infectivity screens in A549-ACE2 knockdown cells (C) and Caco-2 knockout cells (D) sorted by *z*-score (*z* < 0, decreased infectivity; *z* > 0 increased infectivity). Negative controls (nontargeting control for siRNA, nontargeted cells for CRISPR) and positive controls (ACE2 knockdown or knockout) are highlighted. (**E**) Results from both assays with potential hits (|z|>2) highlighted in red (A549-ACE2), yellow (Caco-2), and orange (both). (**F**) Pan-coronavirus interactome reduced to human preys with significant increase (red nodes) or decrease (blue nodes) in SARS-CoV2 replication upon knockdown or knockout. Viral proteins baits from SARS-CoV-2 (red), SARS-CoV-1 (orange), and MERS-CoV (yellow) are represented as diamonds. The thickness of the edge indicates the strength of the PPI in spectral counts. KD, knockdown; KO, knockout; PPI, protein-protein interaction.

To identify host factors that are critical for infection and therefore potential targets for host-directed therapies, we performed genetic perturbations of 332 human proteins—331 previously identified to interact with SARS-CoV-2 proteins (*5*) plus ACE2—and observed their effect on infectivity. To ensure a broad coverage of potential hits, we carried out two screens in different cell lines, investigating the effects on infection: small interfering RNA (siRNA) knockdowns in A549 cells stably expressing ACE2 (A549-ACE2) (Fig. 5A) and CRISPR-based knockouts in Caco-2 cells (Fig. 5B). ACE2 was included as positive control in both screens as were nontargeting siRNAs or nontargeted Caco-2 cells as negative controls. After SARS-CoV-2 infection, effects on virus infectivity were quantified by real-time quantitative polymerase chain reaction (RT-qPCR) on cell supernatants (siRNA) or by titrating virus-containing supernatants on Vero E6 cells (CRISPR) (see Materials and methods for details). Cells were monitored for viability, and knockdown or editing efficiency was determined as described (Materials and methods and fig. S18). This revealed that 93% of the genes were knocked down at least 50% in the A549-ACE2 screen, and 95% of the knockdowns exhibited a <20% decrease in viability. In the Caco-2 assay, we observed an editing efficiency of at least 80% for 89% of the genes tested (Materials and methods and fig. S18). Of the 332 human SARS-CoV-2 interactors, the final A549-ACE2 dataset includes 331 gene knockdowns, and the Caco-2 dataset includes 286 gene knockouts, with the difference mainly owing to the removal of essential genes (Materials and methods). The readouts from both assays were then separately normalized using robust *z*-scores (Materials and methods), with negative and positive *z*-scores indicating proviral-dependency factors (perturbation leads to decreased infectivity) and antiviral host factors with restrictive activity (perturbation leads to increased infectivity), respectively. As expected, negative controls resulted in neutral *z*-scores (Fig. 5, C and D, and tables S6 and S7). Similarly, perturbations of the positive control ACE2 resulted in strongly negative *z*-scores in both assays (Fig. 5, C and D). Overall, the *z*-scores did not exhibit any trends related to viability, knockdown efficiency, or editing efficiency (fig. S18). With a cutoff of |z|>2 to highlight genes that notably affect SARS-CoV-2 infectivity when perturbed, 31 and 40 dependency factors (*z* < −2) and 3 and 4 factors with restrictive activity (*z* > 2) were identified in A549-ACE2 and Caco-2 cells, respectively (Fig. 5E). Of particular interest are the host-dependency factors for SARS-CoV-2 infection, which represent potential targets for drug development and repurposing. For example, nonopioid receptor sigma 1 (sigma-1, encoded by *SIGMAR1*) was identified as a functional host-dependency factor in both cell systems, in agreement with our previous report of antiviral activity for sigma receptor ligands (*5*). To provide a contextual view of the genetics results, we generated a network that integrates the hits from both cell lines and the PPIs of their encoded proteins with SARS-CoV-2, SARS-CoV-1, and MERS-CoV proteins (Fig. 5F). Notably, we observed an enrichment of genetic hits that encode proteins interacting with viral Nsp7, which has a high degree of interactions shared across all three viruses (Fig. 3C). Prostaglandin E synthase 2 (PGES2, encoded by *PTGES2*), for example, is a functional interactor of Nsp7 from SARS-CoV-1, SARS-CoV-2, and MERS-CoV. Other dependency factors were specific to SARS-CoV-2, including interleukin-17 (IL-17) receptor A (*IL17RA*), which interacts with SARS-CoV-2 ORF8. We also identify dependency factors that are shared interactors between SARS-CoV-1 and SARS-CoV-2 such as the aforementioned sigma receptor 1 (*SIGMAR1*), which interacts with Nsp6, and the mitochondrial import receptor subunit Tom70 (*TOMM70*), which interacts with ORF9b. We will use these interactions to validate virus-host interactions (ORF8-IL17RA and ORF9b-Tom70), connect our systems biology data to evidence for the clinical impact of the host factors we identified (IL17RA), and analyze outcomes of COVID-19 patients treated with putative host-directed drugs against PGES-2 and sigma receptor 1.

## SARS ORF9b interacts with Tom70

**Fig. 6:**
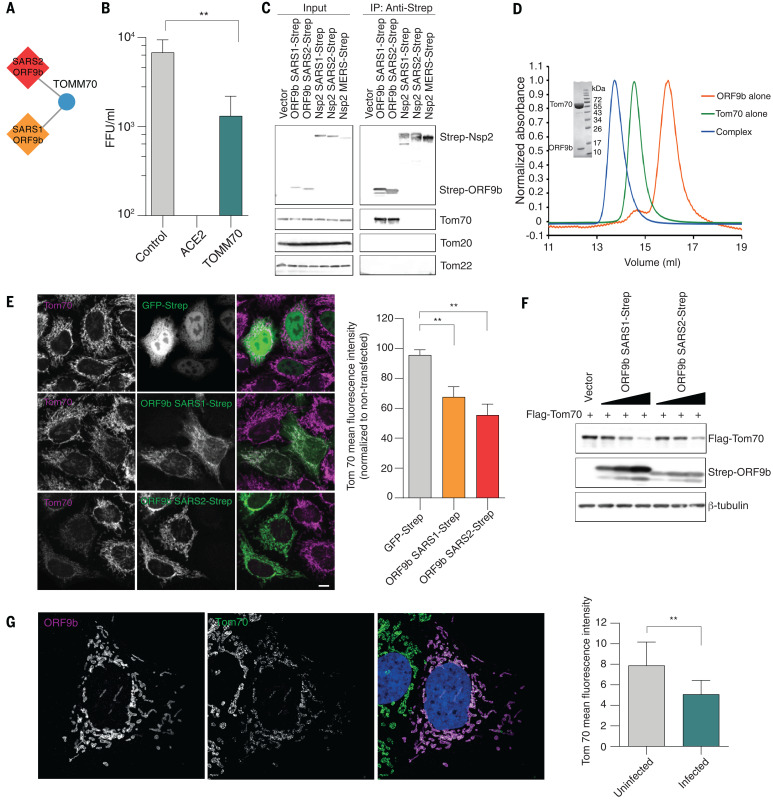
Interaction between ORF9b and human Tom70. (**A**) ORF9b-Tom70 interaction is conserved between SARS-CoV-1 and SARS-CoV-2. (**B**) Viral titers in Caco-2 cells after CRISPR knockout of *TOMM70* or controls. (**C**) Coimmunoprecipitation of endogenous Tom70 with Strep-tagged ORF9b from SARS-CoV-1 and SARS-CoV-2; Nsp2 from SARS-CoV-1, SARS-CoV-2, and MERS-CoV; or vector control in HEK293T cells. Representative blots of whole-cell lysates and eluates after IP are shown. (**D**) Size exclusion chromatography traces (10/300 S200 increase) of ORF9b alone, Tom70 alone, and coexpressed ORF9b-Tom70 complex purified from recombinant expression in *E. coli*. Insert shows SDS-PAGE of the complex peak indicating presence of both proteins. (**E**) Immunostainings for Tom70 in HeLaM cells transfected with GFP-Strep and ORF9b from SARS-CoV-1 and SARS-CoV-2 (left) and mean fluorescence intensity ± SD values of Tom70 in GFP-Strep and ORF9b expressing cells (normalized to nontransfected cells) (right). Scale bar, 10 μm. (**F**) Flag-Tom70 expression levels in total cell lysates of HEK293T cells upon titration of cotransfected Strep-ORF9b from SARS-CoV-1 and SARS-CoV-2. (**G**) Immunostaining for ORF9b and Tom70 in Caco-2 cells infected with SARS-CoV-2 (left) and mean fluorescence intensity ± SD values of Tom70 in uninfected and SARS-CoV-2–infected cells (right). SARS2, SARS-CoV-2; SARS1, SARS-CoV-1; MERS, MERS-CoV; IP, immunoprecipitation. **P < 0.05, Student’s *t* test.

ORF9b of SARS-CoV-1 and SARS-CoV-2 were found to be localized to mitochondria upon overexpression as well as in SARS-CoV-2–infected cells. In line with this, the mitochondrial outer membrane protein Tom70 (encoded by *TOMM70*) is a high-confidence interactor of ORF9b in both SARS-CoV-1 and SARS-CoV-2 interaction maps (Fig. 6A), and it acts as a host-dependency factor for SARS-CoV-2 (Fig. 6B). Tom70 falls below the scoring threshold as a putative interactor of MERS-CoV Nsp2, a viral protein not associated with mitochondria (table S2). Tom70 is one of the major import receptors in the translocase of the outer membrane (TOM) complex that recognizes and mediates the translocation of mitochondrial preproteins from the cytosol into the mitochondria in a chaperone-dependent manner (*8*). Additionally, Tom70 is involved in the activation of the mitochondrial antiviral signaling (MAVS) protein, which leads to apoptosis upon viral infection (*9*, *10*).

To validate the interaction between viral proteins and Tom70, we performed a coimmunoprecipitation experiment in the presence or absence of Strep-tagged ORF9b from SARS-CoV-1 and SARS-CoV-2 as well as Strep-tagged Nsp2 from all three coronaviruses. Endogenous Tom70—but not other translocase proteins of the outer membrane including Tom20, Tom22, and Tom40—coprecipitated only in the presence of ORF9b but not Nsp2 in both HEK293T and A549 cells, which confirms our affinity purification–mass spectrometry (AP-MS) data and suggests that ORF9b specifically interacts with Tom70 (Fig. 6C and fig. S19A). Further, upon coexpression in bacterial cells, we were able to copurify the ORF9b-Tom70 protein complex, which indicates a stable complex (Fig. 6D). We found that SARS-CoV-1 and SARS-CoV-2 ORF9b expressed in HeLaM cells colocalized with Tom70 (Fig. 6E) and observed that SARS-CoV-1 or SARS-CoV-2 ORF9b overexpression led to decreases in Tom70 expression (Fig. 6, E and F). Similarly, ORF9b was found to colocalize with Tom70 on SARS-CoV-2 infection (Fig. 6G). This is in agreement with the known outer mitochondrial membrane localization of Tom70 (*11*) and ORF9b localization to mitochondria upon overexpression and during SARS-CoV-2 infection (Fig. 2B). We also saw decreases in Tom70 expression during SARS-CoV-2 infection (Fig. 6G) but did not see pronounced changes in expression levels of the mitochondrial protein Tom20 after individual Strep-ORF9b expression or upon SARS-CoV-2 infection (fig. S19, B and C).

## Cryo–electron microscopy structure of ORF9b-Tom70 complex reveals ORF9b interacting at the substrate binding site of Tom70

**Fig. 7:**
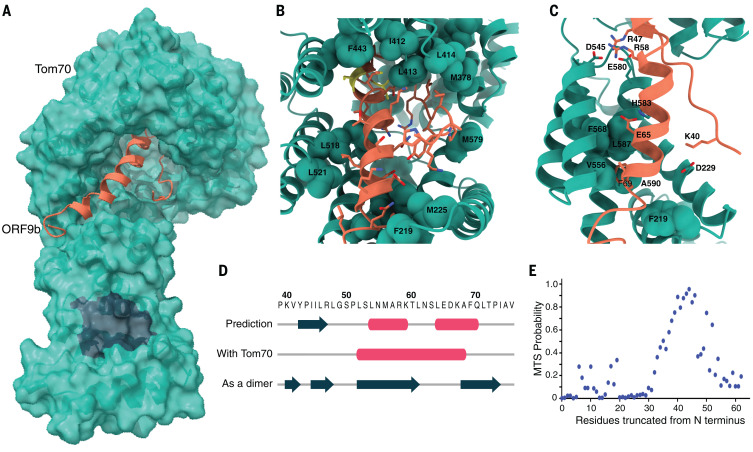
Cryo-EM structure of ORF9b-Tom70 complex reveals ORF9b adopting a helical fold and binding at the substrate recognition site of Tom70. (**A**) Surface representation of the ORF9b-Tom70 structure. Tom70 is depicted as molecular surface in green, ORF9b is depicted as ribbon in orange. Region in charcoal indicates Hsp70 or Hsp90 binding site on Tom70. (**B**) Magnified view of ORF9b-Tom70 interactions with interacting hydrophobic residues on Tom70 indicated and shown in spheres. The two phosphorylation sites on ORF9b, S50 and S53, are shown in yellow. (**C**) Ionic interactions between Tom70 and ORF9b are depicted as sticks. Highly conserved residues on Tom70 making hydrophobic interactions with ORF9b are depicted as spheres. (**D**) Diagram depicting secondary structure comparison of ORF9b as predicted by JPred server—as visualized in our structure—or as visualized in the previously crystallized dimer structure (PDB ID: 6Z4U) (*16*). Pink tubes indicate helices, charcoal arrows indicate beta strands, and the amino acid sequence for the region visualized in the cryo-EM structure is shown on top. (**E**) Predicted probability of having an internal MTS as output by TargetP server by serially running N-terminally truncated regions of SARS-CoV-2 ORF9b. Region visualized in the cryo-EM structure (amino acids 39 to 76) overlaps with the highest internal MTS probability region (amino acids 40 to 50). MTS, mitochondrial targeting signal. Single-letter abbreviations for the amino acid residues are as follows: A, Ala; C, Cys; D, Asp; E, Glu; F, Phe; G, Gly; H, His; I, Ile; K, Lys; L, Leu; M, Met; N, Asn; P, Pro; Q, Gln; R, Arg; S, Ser; T, Thr; V, Val; W, Trp; and Y, Tyr.

Tom70, as part of the TOM complex, is involved in the recognition of mitochondrial preproteins from the cytosol (*12*). To further understand the molecular details of ORF9b-Tom70 interactions, we obtained a 3-Å cryo–electron microscopy (cryo-EM) structure of the ORF9b-Tom70 complex (Fig. 7A and fig. S20). Notably, although purified proteins failed to interact upon attempted in vitro complex reconstitution, they yielded a stable and pure complex when coexpressed in *Escherichia coli* (Fig. 6D). This may be because of the fact that ORF9b alone purifies as a dimer (as inferred by the apparent molecular weight on size-exclusion chromatography) and would need to dissociate to interact with Tom70 on the basis of our structure. Tom70 preferentially binds preproteins with internal hydrophobic targeting sequences (*13*). It contains an N-terminal transmembrane domain and tetratricopeptide repeat (TPR) motifs in its cytosolic segment. The C-terminal TPR motifs recognize the internal mitochondrial targeting signals (MTSs) of preproteins, and the N-terminal TPR clamp domain serves as a docking site for multichaperone complexes that contain preprotein (*14*, *15*). Obtained cryo-EM density allowed us to build atomic models for residues 109 to 600 of human Tom70 and residues 39 to 76 of SARS-CoV-2 ORF9b (Fig. 7A and table S8). ORF9b makes extensive hydrophobic interactions at the pocket on Tom70 that have been implicated in its binding to MTS, with the total buried surface area at the interface being quite extensive—~2000 Å^2^ (Fig. 7B). In addition to the mostly hydrophobic interface, four salt bridges further stabilize the interaction (Fig. 7C). On interaction with ORF9b, the interacting helices on Tom70 move inward to tightly wrap around ORF9b as compared with previously crystallized yeast Tom70 homologs (movie S1). No structure for human Tom70 without a substrate has been reported to date, and therefore we cannot rule out the idea that the conformational differences are because of differences between homologs. However, it is possible that this conformational change upon substrate binding is conserved across homologs, as many of the Tom70 residues interacting with ORF9b are highly conserved, which likely indicates residues essential for endogenous MTS substrate recognition.

Although a previously published crystal structure of SARS-CoV-2 ORF9b revealed that it entirely consists of beta sheets [Protein Data Bank (PDB) ID: 6Z4U] (*16*), we observed that, upon binding Tom70 residues 52 to 68, ORF9b forms a helix (Fig. 7D). This is consistent with the fact that MTS sequences recognized by Tom70 are usually helical, and analysis with the TargetP MTS prediction server revealed a high probability for this region of ORF9b to have an MTS (Fig. 7E). This shows structural plasticity in this viral protein where, depending on the binding partner, ORF9b changes between helical and beta strand folds. Furthermore, we had previously identified two infection-driven phosphorylation sites on ORF9b, S50 and S53 (*17*), which map to the region on ORF9b buried deep in the Tom70 binding pocket (Fig. 7B, yellow). S53 contributes two hydrogen bonds to the interaction with Tom70 in this overall hydrophobic region. Therefore, once phosphorylated, it is likely that the ORF9b-Tom70 interaction is weakened. These residues are surface exposed in the dimeric structure of ORF9b, which could potentially allow phosphorylation to partition ORF9b between Tom70-bound and dimeric populations.

The two binding sites on Tom70—the substrate binding site and the TPR domain that recognizes Hsp70 and Hsp90—are known to be conformationally coupled (*18*). Tom70’s interaction with a C-terminal EEVD motif of Hsp90 via the TPR domain is key for its function in the interferon pathway and induction of apoptosis on viral infection (*10*, *19*). Whether ORF9b, by binding to the substrate recognition site of Tom70, allosterically inhibits Tom70’s interaction with Hsp90 at the TPR domain remains to be investigated; but notably we observe in our structure that R192, a key residue in the interaction with Hsp70 and Hsp90, is moved out of position to interact with the EEVD sequence, which suggests that ORF9b may modulate interferon and apoptosis signaling via Tom70 (fig. S21). Alternatively, Tom70 has been described as an essential import receptor for PTEN induced kinase 1 (PINK1), and therefore the loss of mitochondrial import efficiency as a result of ORF9b binding to the Tom70 substrate binding pocket may induce mitophagy.

## Implications of the ORF8-IL17RA interaction for COVID-19

**Fig. 8:**
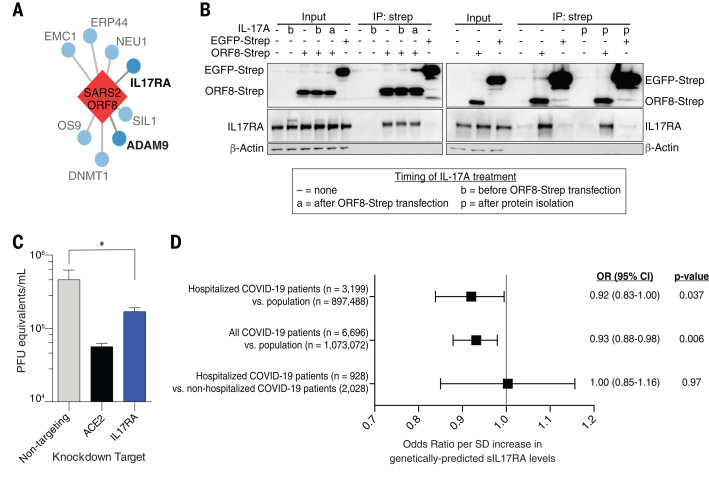
SARS-CoV-2 ORF8 and functional interactor IL17RA are linked to viral outcomes. (**A**) IL17RA and ADAM9 are functional interactors of SARS-CoV-2 ORF8. Only interactors identified in the genetic screening are shown. (**B**) Coimmunoprecipitation of endogenous IL17RA with Strep-tagged ORF8 or EGFP with or without IL-17A treatment at different times. Overexpression was done in HEK293T cells. (**C**) Viral titer after *IL17RA* or control knockdown in A549-ACE2 cells. (**D**) OR of membership in indicated cohorts by genetically predicted sIL17RA levels. SARS2, SARS-CoV-2; IP, immunoprecipitation; SD, standard deviation; OR, odds ratio; CI, confidence interval; sIL17RA, soluble IL17RA. *P < 0.05, unpaired *t* test. Error bars in (C) indicate SDs; in (D), they indicate 95% CIs.

As described above, we found that IL-17 receptor A (IL17RA) physically interacts with ORF8 from SARS-CoV-2, but not SARS-CoV-1 or MERS-CoV (Fig. 5D, table S2, and Fig. 8A). Several recent studies have identified high IL-17 levels or aberrant IL-17 signaling as a correlate of severe COVID-19 (*20*–*23*). We demonstrated that the physical interaction of SARS-CoV-2 ORF8 with IL17RA occurs with or without IL-17A treatment, which suggests that signaling through the receptor does not disrupt the interaction with ORF8 (Fig. 8B). Furthermore, knockdown of IL17RA led to a significant decrease in SARS-CoV-2 viral replication in A549-ACE2 cells (Fig. 8C). These data suggest that the ORF8-IL17RA interaction modulates systemic IL-17 signaling.

One manner in which this signaling is regulated is through the release of the extracellular domain of the receptor as soluble IL17RA (sIL17RA), which acts as a decoy in circulation by soaking up IL-17A and inhibiting IL-17 signaling (*24*). Production of sIL17RA has been demonstrated by alternative splicing in cultured cells (*25*), but the mechanism by which IL17RA is shed in vivo remains unclear (*26*). ADAM family metalloproteases are known to mediate the release of other interleukin receptors into their soluble form (*27*). We found that SARS-CoV-2 ORF8 physically interacted with both ADAM9 and ADAMTS1 in our previous study (*5*). We find that knockdown of ADAM9, like that of IL17RA, leads to significant decreases in SARS-CoV-2 replication in A549-ACE2 cells (Fig. 5D and table S2).

To test the in vivo relevance of sIL17RA in modulating SARS-CoV-2 infection, we leveraged a genome-wide association study (GWAS) which identified 14 single-nucleotide polymorphisms (SNPs) near the *IL17RA* gene that causally regulate sIL17RA plasma levels (*28*). We then used generalized summary-based Mendelian randomization (GSMR) (*28*, *29*) on the curated GWAS datasets of the COVID-19 Host Genetics Initiative (COVID-HGI) (*30*) and observed that genotypes that predicted higher sIL17RA plasma levels were associated with lower risk of COVID-19 when compared with the population (Fig. 8D and table S9), which is seemingly consistent with our molecular data. Similar results were obtained when comparing only hospitalized COVID-19 patients to the population. However, there was no evidence of association in hospitalized versus nonhospitalized COVID-19 patients. Though the COVID-HGI dataset is underpowered and this observation needs to be replicated in other cohorts, the clinical observations, functional genetics, and clinical genetics all suggest that SARS-CoV-2 benefits from modulating IL-17 signaling. One potentially contradictory caveat is that we find high-level IL-17A treatment diminishes SARS-CoV-2 replication in A549-ACE2 cells (fig. S22); however, IL-17 is a pleiotropic cytokine and it is likely to play multiple roles during SARS-CoV-2 infection in the context of a competent immune system.

Infectious and transmissible SARS-CoV-2 viruses with large deletions of ORF8 have arisen during the pandemic and have been associated with milder disease and lower concentrations of proinflammatory cytokines (*31*). Notably, compared with healthy controls, patients infected with wildtype, but not ORF8-deleted virus, had threefold elevated plasma levels of IL-17A (*31*). More work will be needed to understand if and how ORF8 manipulates the IL-17 signaling pathway during the course of SARS-CoV-2 infection.

## Investigation of druggable targets identified as interactors of multiple coronaviruses

The identification of druggable host factors provides a rationale for drug repurposing efforts. Given the extent of the current pandemic, real-world data can now be used to study the outcome of COVID-19 patients coincidentally treated with host factor–directed, U.S. Food and Drug Administration (FDA)–approved therapeutics. Using medical billing data, we identified 738,933 patients in the United States with documented SARS-CoV-2 infection (Materials and methods). In this cohort, we probed the use of drugs against targets identified here that were shared across coronavirus strains and found to be functionally relevant in the genetic perturbation screens. In particular, we analyzed outcomes for an inhibitor of prostaglandin E synthase type 2 (PGES-2, encoded by *PTGES2*) and for potential ligands of sigma nonopioid receptor 1 (sigma-1, encoded by *SIGMAR1*), and investigated whether these patients fared better than carefully matched patients treated with clinically similar drugs without predicted anticoronavirus activity.

**Fig. 9:**
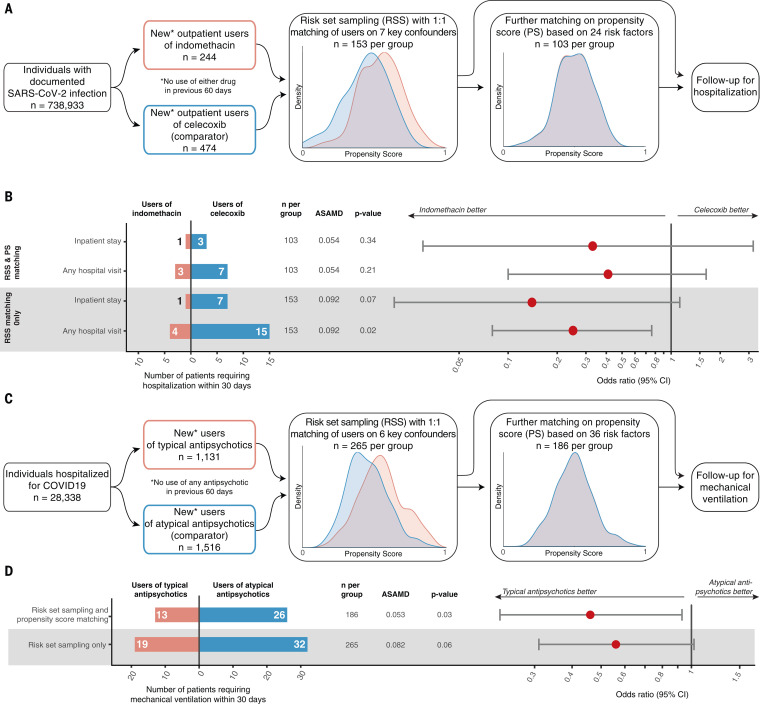
Real-world data analysis of drugs identified through molecular investigation support their antiviral activity. (**A**) Schematic of retrospective real-world clinical data analysis of indomethacin use for outpatients with SARS-CoV-2. Plots show distribution of propensity scores (PSs) for all included patients (red, indomethacin users; blue, celecoxib users). For a full list of inclusion, exclusion, and matching criteria, see Materials and methods and table S11. (**B**) Effectiveness of indomethacin versus celecoxib in patients with confirmed SARS-CoV-2 infection treated in an outpatient setting. Average standardized absolute mean difference (ASAMD) is a measure of balance between indomethacin and celecoxib groups calculated as the mean of the absolute standardized difference for each PS factor (table S11); *P* value and ORs with 95% CIs are estimated using the Aetion Evidence Platform r4.6. No ASAMD was >0.1. (**C**) Schematic of retrospective real-world clinical data analysis of typical antipsychotic use for inpatients with SARS-CoV-2. Plots show distribution of PSs for all included patients (red, typical users; blue, atypical users). For a full list of inclusion, exclusion, and matching criteria see Materials and methods and table S11. (**D**) Effectiveness of typical versus atypical antipsychotics among hospitalized patients with confirmed SARS-CoV-2 infection treated in hospital. ASAMD is a measure of balance between typical and atypical groups calculated as the mean of the absolute standardized difference for each PS factor (table S11); *P* value and ORs with 95% CIs are estimated using the Aetion Evidence Platform r4.6. No ASAMD was >0.1.

PGES-2, an interactor of Nsp7 from all three viruses (Fig. 4D), is a dependency factor for SARS-CoV-2 (Fig. 5F). It is inhibited by the FDA-approved prescription nonsteroidal anti-inflammatory drug (NSAID) indomethacin. Computational docking of Nsp7 and PGES-2 to predict binding configuration showed that the dominant cluster of models localizes Nsp7 adjacent to the PGES-2–indomethacin binding site (fig. S23). However, indomethacin did not inhibit SARS-CoV-2 in vitro at reasonable antiviral concentrations (fig. S24 and table S10). A previous study also found that similarly high levels of the drug were needed for inhibition of SARS-CoV-1 in vitro, but this study still showed efficacy for indomethacin against canine coronavirus in vivo (*32*). This motivated us to observe outcomes in a cohort of outpatients with confirmed SARS-CoV-2 infection who by happenstance initiated a course of indomethacin compared with those who initiated the prescription NSAID celecoxib, which lacks anti–PGES-2 activity. We compared the odds of hospitalization by risk-set sampling (RSS) patients treated at the same time and at similar levels of disease severity and then by further matching on propensity score (PS) (*33*) (Fig. 9A and table S11). RSS and PS—combined with a new user, active comparator design that mimics the interventional component of parallel group randomized studies—are established design and analytic techniques that mitigate biases that can arise in observational studies. A complete list of risk factors used for matching, which include demographic data, baseline health care utilization, comorbidities, and measures of disease severity, are found in table S11.

Among SARS-CoV-2–positive patients, new users of indomethacin in the outpatient setting were less likely than matched new users of celecoxib to require hospitalization or inpatient services [Fig. 9B; odds ratio (OR) = 0.33; 95% confidence interval (CI): 0.03 to 3.19]. The CI of our primary analysis included the null value. In sensitivity analyses, neither using the larger, risk-set–sampled cohort nor relaxing our outcome definition to include any hospital visit appreciably changed the interpretation of our findings, but it did narrow the CIs, particularly when both approaches were combined (OR = 0.25; 95% CI: 0.08 to 0.76). Although we acknowledge that this is a small, noninterventional study, it is nonetheless an example of how molecular insight can rapidly generate testable clinical hypotheses and help prioritize candidates for prospective clinical trials or future drug development.

To create larger patient cohorts, we next grouped drugs that shared activity against the same target—sigma receptors. We previously identified sigma-1 and sigma-2 as drug targets in our SARS-CoV-2–human PPI map, and multiple potent, nonselective sigma ligands were among the most promising inhibitors of SARS-CoV-2 replication in Vero E6 cells (*5*). As shown above, knockout and knockdown of *SIGMAR1*, but not of *SIGMAR2* (also known as *TMEM97*), led to robust decreases in SARS-CoV-2 replication (fig. S24 and Fig. 5F), which suggests that sigma-1 may be a key therapeutic target. We analyzed *SIGMAR1* sequences across 359 mammals and observed positive selection of several residues within beaked whale, mouse, and ruminant lineages, which may indicate a role in host-pathogen competition (fig. S25). Additionally, the sigma ligand drug amiodarone inhibited replication of SARS-CoV-1 as well as SARS-CoV-2, consistent with the conservation of the Nsp6–sigma-1 interaction across the SARS viruses (fig. S24 and Fig. 4D). We then looked for other FDA-approved drugs with reported nanomolar affinity for sigma receptors or those that fit the sigma ligand chemotype (*5*, *34*–*41*), and we selected 13 such therapeutics. We find that all are potent inhibitors of SARS-CoV-2 with half-maximal inhibitory concentration (IC_50_) values <10 μM, though there is a wide range in reported sigma receptor affinity with no clear correlation between sigma receptor binding affinity and antiviral activity (fig. S24D). Several clinical drug classes were represented by more than one candidate, including typical antipsychotics and antihistamines. Over-the-counter antihistamines are not well represented in medical billing data and are therefore poor candidates for real-world analysis, but users of typical antipsychotics can be easily identified in our patient cohort. By grouping these individual drug candidates by clinical indication, we were able to build a better-powered comparison.

We constructed a cohort for retrospective analysis on new, inpatient users of antipsychotics. In inpatient settings, typical and atypical antipsychotics are used similarly, most commonly for delirium. We compared the effectiveness of typical antipsychotics, which have sigma activity and antiviral effects (fig. S24E), versus atypical antipsychotics, which do not have antiviral activity (fig. S24F), for treatment of COVID-19 (Fig. 9C). Observing mechanical ventilation outcomes in inpatient cohorts is a proxy for the worsening of severe illness rather than the progression from mild disease signified by the hospitalization of indomethacin-exposed outpatients above. We again used RSS plus PS to build a robust, directly comparable cohort of inpatients (table S11). In our primary analysis, half as many of the new users of typical antipsychotics compared with the new users of atypical antipsychotics progressed to the point of requiring mechanical ventilation, demonstrating significantly lower use with an OR of 0.46 (95% CI: 0.23 to 0.93; *P* = 0.03; Fig. 9D). As above, we conducted a sensitivity analysis in the RSS-only cohort and observed the same trend (OR = 0.56; 95% CI: 0.31 to 1.02; *P* = 0.06), which emphasizes the primary result of a beneficial effect for typical versus atypical antipsychotics observed in the RSS-plus-PS–matched cohort. Although a careful analysis of the relative benefits and risks of typical antipsychotics should be undertaken before considering prospective studies or interventions, these data and analyses demonstrate how molecular information can be translated into real-world implications for the treatment of COVID-19—an approach that can ultimately be applied to other diseases in the future.

## Discussion

In this study, we generated and compared three different coronavirus-human PPI maps in an attempt to identify and understand pan-coronavirus molecular mechanisms. The use of a quantitative DIS allowed for the identification of virus-specific as well as shared interactions among distinct coronaviruses. We also systematically carried out subcellular localization analysis using tagged viral proteins and antibodies targeting specific SARS-CoV-2 proteins. Our results suggest that protein localization can often differ when comparing individually expressed viral proteins with the localization of the same protein in the context of infection. This can be because of factors such as mislocation driven by tagging, changes in localization due to interaction partners, or cellular compartments that are specific to the infection state. These differences are notable caveats of virus-host interaction studies performed with tagged, expressed proteins. However, previous studies and the work performed here show how these data can be powerful for the identification of host-targeted processes and relevant drug targets.

These data were integrated with genetic data where the interactions uncovered with SARS-CoV-2 were perturbed using RNA interference (RNAi) and CRISPR in different cellular systems and viral assays—an effort that functionally connected many host factors to infection. One of these, Tom70, which we have shown binds to ORF9b from both SARS-CoV-1 and SARS-CoV-2, is a mitochondrial outer membrane translocase that has been previously shown to be important for mounting an interferon response (*42*). Our functional data, however, show that Tom70 has at least some role in promoting infection rather than inhibiting it. Using cryo-EM, we obtained a 3-Å structure of a region of ORF9b binding to the active site of Tom70. Notably, we found that ORF9b is in a markedly different conformation than previously visualized. This suggests the possibility that ORF9b may partition between two distinct structural states, with each having a different function and possibly explaining its apparent pleiotropy. The exact details of functional significance and regulation of the ORF9b-Tom70 interaction will require further experimental elucidation. This interaction, however, which is conserved between SARS-CoV-1 and SARS-CoV-2, could have value as a pan-coronavirus therapeutic target.

Finally, we attempted to connect our in vitro molecular data to clinical information available for COVID-19 patients to understand the pathophysiology of COVID-19 and explore therapeutic avenues. To this end, using GWAS datasets of the COVID-HGI (*30*), we observed that increased predicted sIL17RA plasma levels were associated with lower risk of COVID-19. Notably, we find that IL17RA physically binds to SARS-CoV-2 ORF8, and genetic disruption results in decreased infection. These collective data suggest that future studies should be focused on this pathway as both an indicator and therapeutic target for COVID-19. Furthermore, using medical billing data, we also observed trends in COVID-19 patients on specific drugs indicated by our molecular studies. For example, inpatients prescribed sigma-ligand typical antipsychotics appear to have better COVID-19 outcomes compared with users of atypical antipsychotics, which do not have anti–SARS-CoV-2 activity in vitro. However, we cannot be certain that the sigma receptor interaction is the mechanism underpinning this effect, as typical antipsychotics are known to bind to a multitude of cellular targets, and some atypical antipsychotics, which lack anti–SARS-CoV-2 activity, nonetheless have reported affinity for rodent sigma receptors (table S10). Replication in other patient cohorts and further work will be needed to see whether there is therapeutic value in these connections, but we have at least demonstrated a strategy wherein protein network analyses can be used to make testable predictions from real-world clinical information.

We have described an integrative and collaborative approach to study and understand pathogenic coronavirus infection, identifying conserved targeted mechanisms that are likely to be of high relevance for other viruses of this family, some of which have yet to infect humans. We used proteomics, cell biology, virology, genetics, structural biology, biochemistry, and clinical and genomic information in an attempt to provide a holistic view of SARS-CoV-2 and other coronaviruses’ interactions with infected host cells. We propose that such an integrative and collaborative approach could and should be used to study other infectious agents as well as other disease areas.

## Materials and methods

### Cells

HEK293T/17 (HEK293T) cells were procured from the University of California, San Francisco (UCSF) Cell Culture Facility, and are available through UCSF's Cell and Genome Engineering Core (https://cgec.ucsf.edu/cell-culture-and-banking-services). HEK293T cells were cultured in Dulbecco’s modified Eagle’s medium (DMEM) (Corning) supplemented with 10% fetal bovine serum (FBS) (Gibco, Life Technologies) and 1% penicillin-streptomycin (Corning) and maintained at 37°C in a humidified atmosphere of 5% CO_2_. Short tandem repeat (STR) analysis by the Berkeley Cell Culture Facility on 8 August 2017 authenticates these as HEK293T cells with 94% probability.

HeLaM cells (RRID: CVCL_R965) were originally obtained from the laboratory of M. S. Robinson (CIMR, University of Cambridge, UK) and have been routinely tested for mycoplasma contamination. HeLaM cells were grown in DMEM supplemented with 10% FBS, 100 U/ml penicillin, 100 μg/ml streptomycin, and 2 mM glutamine at 37°C in a 5% CO_2_ humidified incubator.

A549 cells stably expressing ACE2 (A549-ACE2) were a gift from O. Schwartz. A549-ACE2 cells were cultured in DMEM supplemented with 10% FBS, blasticidin (20 μg/ml) (Sigma) and maintained at 37°C with 5% CO_2_. STR analysis by the Berkeley Cell Culture Facility on 17 July 2020 authenticates these as A549 cells with 100% probability.

Caco-2 cells (ATTC, HTB-37, RRID:CVCL_0025) were cultured in DMEM with GlutaMAX and pyruvate (Gibco, 10569010) and supplemented with 20% FBS (Gibco, 26140079). For Caco-2 cells utilized in Cas9-RNP knockouts, STR analysis by the Berkeley Cell Culture Facility on 23 April 2020 authenticates these as Caco-2 cells with 100% probability.

Vero E6 cells were purchased from the American Type Culture Collection (ATCC) and thus authenticated [VERO C1008 (Vero 76, clone E6, Vero E6)] (ATCC, CRL-1586). Vero E6 cells tested negative for mycoplasma contamination. Vero E6 cells were cultured in DMEM (Corning) supplemented with 10% FBS (Gibco, Life Technologies) and 1% penicillin-streptomycin (Corning) and maintained at 37°C in a humidified atmosphere of 5% CO_2_.

### Microbes

LOBSTER *E. coli* Expression Strain: LOBSTR-[BL21(DE3)] Kerafast no. EC1002.

### Antibodies

#### Commercially available primary antibodies used in this study

Rabbit anti-beta-actin (Cell Signaling Technology #4967, RRID:AB_330288); mouse anti-beta tubulin (Sigma-Aldrich #T8328, RRID:AB_1844090); rabbit anti-BiP (Cell Signaling Technology #3177S, RRID:AB_2119845); mouse anti-EEA1 (BD Biosciences #610457, RRID:AB_397830, used at 1:200); mouse anti-ERGIC53 (Enzo Life Sciences #ALX-804-602-C100, RRID:AB_2051363, used at 1:200); anti-GM130; rabbit anti-GRP78 BiP (Abcam #Ab21685, RRID:AB_2119834); rabbit anti–SARS-CoV–nucleocapsid protein (NP) (Rockland #200-401-A50, RRID:AB_828403); rabbit anti-PDI (protein disulfide isomerase) (Cell Signaling Technology #3501, RRID:AB_2156433); mouse anti-Strep tag (QIAGEN #34850, RRID:AB_2810987, used at 1:5000); mouse anti-strepMAB (IBA Lifesciences #2-1507-001, used at 1:1000); rabbit anti–Strep-tag II (Abcam #ab232586); rabbit anti-Tom20 (Proteintech #11802-1-AP, RRID:AB_2207530, used at 1:1000); rabbit anti-Tom20 (Cell Signaling Technology #42406, RRID:AB_2687663); mouse anti-Tom22 (Santa Cruz Biotechnology #sc-101286, RRID:AB_1130526); rabbit anti-Tom40 (Santa Cruz Biotechnology #sc-11414, RRID:AB_793274); mouse anti-Tom70 (Santa Cruz #sc-390545, RRID:AB_2714192, used at 1:500); Rabbit anti-STX5 (Synaptic Systems 110 053, used at 1:500); and ActinStaining Kit 647-Phalloidin (Hypernol #8817-01, used at 1:400).

#### Commercially available secondary antibodies used in this study

Alexa Fluor 488 chicken anti-mouse immunoglobulin G (IgG) (Invitrogen #A21200, RRID_AB_2535786, used at 1:400); Alexa Fluor 488 chicken anti-rabbit IgG (Invitrogen #A21441, RRID_AB_10563745, used at 1:400); Alexa Fluor 568 donkey anti-sheep IgG (Invitrogen #A21099, RRID_AB_10055702, used at 1:400); Alexa Fluor Plus 488 goat anti-rabbit (ThermoFisher A32731, used at 1:500); Alexa Fluor Plus 594 goat anti-mouse (ThermoFisher A32742, used at 1:500); and goat anti-mouse IgG-HRP (horseradish peroxidase) (BioRad #170-6516, RRID:AB_11125547, used at 1:20000).

#### Noncommercial antisera

Rabbit anti–SARS-CoV-2–NP antiserum was produced by the Garcia-Sastre laboratory and used at 1:10000. For information on polyclonal sheep antibodies targeting SARS-CoV-2 proteins, see below, table S3, and https://mrcppu-covid.bio/.

### Coronavirus annotation and plasmid cloning

SARS-CoV-1 isolate Tor2 (NC_004718) and MERS-CoV (NC_019843) were downloaded from GenBank and utilized to design 2xStrep-tagged expression constructs of ORFs and proteolytically mature Nsps derived from ORF1ab (with N-terminal methionines and stop codons added as necessary). Protein termini were analyzed for predicted acylation motifs, signal peptides, and transmembrane regions, and either the N or C terminus was chosen for tagging as appropriate. Finally, reading frames were codon optimized and cloned into pLVX-EF1alpha-IRES-Puro (Takara/Clontech) including a 5′ Kozak motif.

### Immunofluorescence microscopy of viral protein constructs

Approximately 60,000 HeLaM cells were seeded onto glass coverslips in a 12-well dish and grown overnight. The cells were transfected using 0.5 μg of plasmid DNA and either polyethylenimine (Polysciences) or Fugene HD (Promega; 1 part DNA to 3 parts transfection reagent) and grown for a further 16 hours.

Transfected cells were fixed with 4% paraformaldehyde (Polysciences) in phosphate-buffered saline (PBS) at room temperature for 15 min. The fixative was removed and quenched using 0.1 M glycine in PBS. The cells were permeabilized using 0.1% saponin in PBS containing 10% FBS. The cells were stained with the indicated primary and secondary antibodies for 1 hour at room temperature. The coverslips were mounted onto microscope slides using ProLong Gold antifade reagent (ThermoFisher) and imaged using a UplanApo 60x oil (NA 1.4) immersion objective on a Olympus BX61 motorized wide-field epifluorescence microscope. Images were captured using a Hamamatsu Orca monochrome camera and processed using ImageJ.

To gain insight into the intracellular distribution of each Strep-tagged construct, ~100 cells per transfection were manually scored. Each construct was assigned an intracellular distribution in relation to the plasma membrane, ER, Golgi, cytoplasm, and mitochondria (scored out of 7). Many of the constructs had several localizations so this was also reflected in the scoring. The scoring also took into account the impact of expression level on the localization of the constructs.

### Meta-analysis of immunofluorescence data

We first sorted the data concerning viral protein location for all Strep-tagged viral proteins expressed individually in three heatmaps (one per virus) using a custom R script (“pheatmap” package). The information concerning protein localization during SARS-CoV-2 infection was added as a square border color code in the first heatmap, to compare the two different localization patterns. To compare the predicted versus the experimentally determined locations, for each protein we took the top scoring sequence-based localization prediction from DeepLoc (*43*) if the score was >1. When more than one localization can be assigned to the same protein, we took as many top scoring ones as experimentally assigned localizations we had for the same protein. Finally, for each cell compartment, we count the number of experimentally assigned viral proteins and the subset of them predicted to that same compartment as correct predictions. To compare changes in protein interactions with changes in protein localization (Strep-tagged experiment versus sequence-based prediction), we calculated the Jaccard index of prey overlap for each viral protein (SARS-CoV-2 versus SARS-CoV-1 and SARS-CoV-2 versus MERS-CoV) and plotted them together, for proteins with the same localization and for proteins with different localization.

### Generation of polyclonal sheep antibodies targeting SARS-CoV-2 proteins

Sheep were immunized with individual N-terminal glutathione *S*-transferase (GST)–tagged SARS-CoV-2 recombinant proteins or N-terminal maltose binding protein (MBP)–tagged proteins (for SARS-CoV-2 S, S-RBD, and ORF7a), followed by up to five booster injections 4 weeks apart from each other. Sheep were subsequently bled and IgGs were affinity purified using the specific recombinant N-terminal MBP-tagged viral proteins. Each antiserum specifically recognized the appropriate native viral protein. Characterization of each antibody can be found at https://mrcppu-covid.bio/. All antibodies generated can be requested at https://mrcppu-covid.bio/. Also see table S3.

### Immunofluorescence microscopy of infected Caco-2 cells

For infection experiments in human colon epithelial Caco-2 cells (ATCC, HTB-37), SARS-CoV-2 isolate Muc-IMB-1 (provided by the Bundeswehr Institute of Microbiology, Munich, Germany) was used. SARS-CoV-2 was propagated in Vero E6 cells in DMEM supplemented with 2% FBS. All work involving live SARS-CoV-2 was performed in the BSL3 facility of the Institute of Virology, University Hospital Freiburg, and was approved according to the German Act of Genetic Engineering by the local authority (Regierungspraesidium Tuebingen, permit UNI.FRK.05.16/05).

Caco-2 human colon epithelial cells seeded on glass coverslips were infected with SARS-CoV-2 {Strain Muc-IMB-1/2020, second passage on Vero E6 cells [2 × 10^6^ plaque-forming units (PFU)/ml]} at a multiplicity of infection (MOI) of 0.1. At 24 hours postinfection, cells were washed with PBS and fixed in 4% paraformaldehyde in PBS for 20 min at room temperature, followed by 5 min of quenching in 0.1 M glycine in PBS at room temperature. Cells were permeabilized and blocked in 0.1% saponin in PBS supplemented with 10% FBS for 45 min at room temperature and incubated with primary antibodies for 1 hour at room temperature. After washing 15 min with blocking solution, AF568-labeled donkey–anti-sheep (Invitrogen, #A21099; 1:400) secondary antibody as well as AF4647-labeled Phalloidin (Hypermol, #8817-01; 1:400) were applied for 1 hour at room temperature. Subsequent washing was followed by embedding in Diamond Antifade Mountant with 4′,6-diamidino-2-phenylindole (DAPI) (ThermoFisher, #P36971). Fluorescence images were generated using a LSM800 confocal laser-scanning microscope (Zeiss) equipped with a 63X, 1.4 NA oil objective and Airyscan detector and the Zen blue software (Zeiss) and processed with Zen blue software and ImageJ/Fiji.

### Transfection and cell harvest for immunoprecipitation experiments

For each affinity purification [SARS-CoV-1 baits, MERS-CoV baits, green fluorescent protein (GFP)–2xStrep, or empty vector controls], 10 million HEK293T cells were transfected with up to 15 μg of individual expression constructs using PolyJet transfection reagent (SignaGen Laboratories) at a 1:3 μg:μl ratio of plasmid to transfection reagent on the basis of the manufacturer’s protocol. After >38 hours, cells were dissociated at room temperature using 10 ml PBS without calcium and magnesium (D-PBS) with 10 mM ethylenediaminetetraacetic acid (EDTA) for at least 5 min, pelleted by centrifugation at 200 × *g*, at 4°C for 5 min, washed with 10 ml D-PBS, pelleted once more, and frozen on dry ice before storage at −80°C for later immunoprecipitation analysis. For each bait, three independent biological replicates were prepared.

Whole-cell lysates were resolved on 4 to 20% Criterion SDS–polyacrylamide gel electrophoresis (SDS-PAGE) gels (Bio-Rad Laboratories) to assess Strep-tagged protein expression by immunoblotting using mouse anti-Strep tag antibody 34850 (QIAGEN) and anti-mouse HRP secondary antibody (BioRad).

### Anti–Strep tag affinity purification

Frozen cell pellets were thawed on ice for 15 to 20 min and suspended in 1 ml lysis buffer [immunoprecipitation (IP) buffer (50 mM tris-HCl, pH 7.4 at 4°C; 150 mM NaCl, 1 mM EDTA) supplemented with 0.5% Nonidet P 40 Substitute (NP-40; Fluka Analytical) and cOmplete mini EDTA-free protease and PhosSTOP phosphatase inhibitor cocktails (Roche)]. Samples were then freeze-fractured by refreezing on dry ice for 10 to 20 min, then rethawed and incubated on a tube rotator for 30 min at 4°C. Debris was pelleted by centrifugation at 13,000 × *g*, at 4°C for 15 min. Up to 56 samples were arrayed into a 96-well Deepwell plate for affinity purification on the KingFisher Flex Purification System (Thermo Scientific) as follows: MagStrep “type3” beads (30 μl; IBA Lifesciences) were equilibrated twice with 1 ml wash buffer (IP buffer supplemented with 0.05% NP-40) and incubated with 0.95 ml lysate for 2 hours. Beads were washed three times with 1 ml wash buffer and then once with 1 ml IP buffer. Beads were released into 75 μl denaturation-reduction buffer [2 M urea, 50 mM Tris-HCl pH 8.0, 1 mM dithiothreitol (DTT)] in advance of on-bead digestion. All automated protocol steps were performed at 4°C using the slow mix speed and the following mix times: 30 s for equilibration and wash steps, 2 hours for binding, and 1 min for final bead release. Three 10-s bead collection times were used between all steps.

### On-bead digestion for affinity purification

Bead-bound proteins were denatured and reduced at 37°C for 30 min, alkylated in the dark with 3 mM iodoacetamide for 45 min at room temperature, and quenched with 3 mM DTT for 10 min. To offset evaporation, 22.5 μl 50 mM Tris-HCl, pH 8.0 were added before trypsin digestion. Proteins were then incubated at 37°C, initially for 4 hours with 1.5 μl trypsin (0.5 μg/μl; Promega) and then another 1 to 2 hours with 0.5 μl additional trypsin. All steps were performed with constant shaking at 1100 rpm on a ThermoMixer C incubator. Resulting peptides were combined with 50 μl 50 mM Tris-HCl, pH 8.0 used to rinse beads and acidified with trifluoroacetic acid (0.5% final, pH < 2.0). Acidified peptides were desalted for MS analysis using a BioPureSPE Mini 96-Well Plate (20 mg PROTO 300 C18; The Nest Group, Inc.) according to standard protocols.

### MS operation and peptide search

Samples were resuspended in 4% formic acid, 2% acetonitrile solution, and separated by a reversed-phase gradient over a nanoflow C18 column (Dr. Maisch). HPLC buffer A was composed of 0.1% formic acid, and HPLC buffer B was composed of 80% acetonitrile in 0.1% formic acid. Peptides were eluted by a linear gradient from 7 to 36% B over the course of 52 min, after which the column was washed with 95% B and re-equilibrated at 2% B. Each sample was directly injected by means of an Easy-nLC 1200 (Thermo Fisher Scientific) into a Q-Exactive Plus mass spectrometer (Thermo Fisher Scientific) and analyzed with a 75-min acquisition, with all MS1 and MS2 spectra collected in the orbitrap; data were acquired using the Thermo software Xcalibur (4.2.47) and Tune (2.11 QF1 Build 3006). For all acquisitions, QCloud was used to control instrument longitudinal performance during the project (*44*). All proteomic data were searched against the human proteome (uniprot reviewed sequences downloaded 28 February 2020), enhanced green fluorescent protein (EGFP) sequence, and the SARS-CoV or MERS protein sequences using the default settings for MaxQuant (version 1.6.12.0) (*45*). Detected peptides and proteins were filtered to 1% false discovery rate in MaxQuant. All MS raw data and search results files have been deposited to the ProteomeXchange Consortium via the PRIDE partner repository with the dataset (PXD identifier PXD021588).

### High-confidence protein interaction scoring

Identified proteins were then subjected to PPI scoring with both SAINTexpress (version 3.6.3) and MiST (https://github.com/kroganlab/mist) (*6*, *7*). We applied a two-step filtering strategy to determine the final list of reported interactors, which relied on two different scoring stringency cut-offs. In the first step, we chose all protein interactions that had a MiST score ≥0.7, a SAINTexpress Bayesian false-discovery rate (BFDR) ≤0.05, and an average spectral count ≥2. For all proteins that fulfilled these criteria, we extracted information about the stable protein complexes that they participated in from the CORUM (*46*) database of known protein complexes. In the second step, we then relaxed the stringency and recovered additional interactors that (i) formed complexes with interactors determined in filtering step 1 and (ii) fulfilled the following criteria: MiST score ≥0.6, SAINTexpress BFDR ≤0.05, and average spectral counts ≥2. Proteins that fulfilled filtering criteria in either step 1 or step 2 were considered to be high-confidence protein-protein interactions (HC-PPIs).

Using this filtering criteria, nearly all of our baits recovered a number of HC-PPIs in close alignment with previous datasets reporting an average of around six PPIs per bait (*47*). However, for a subset of baits, we observed a much higher number of PPIs that passed these filtering criteria. For these baits, the MiST scoring was instead performed using a larger in-house database of 87 baits that were prepared and processed in an analogous manner to this SARS-CoV-2 dataset. This was done to provide a more comprehensive collection of baits for comparison, to minimize the classification of nonspecifically binding background proteins as HC-PPIs. This was performed for SARS-CoV-1 baits (M, Nsp12, Nsp13, Nsp8, and ORF7b), MERS-CoV baits (Nsp13, Nsp2, and ORF4a), and SARS-CoV-2 Nsp16. SARS-CoV-2 Nsp16 MiST was scored using the in-house database as well as all previous SARS-CoV-2 data (*5*).

### Hierarchical clustering of virus-human protein interactions

Hierarchical clustering was performed on interactions for (i) viral bait proteins shared across all three viruses and (ii) passed the high-confidence scoring criteria (MiST score ≥0.6, SAINTexpress BFDR ≤0.05, and average spectral counts ≥2) in at least one virus. We clustered using a new interaction score (*K*), which we defined as the average between the MiST and SAINT score for each virus-human interaction. This was done to provide a single score that captured the benefits from each scoring method. Clustering was performed using the ComplexHeatmap package in R, using the “average” clustering method and “euclidean” distance metric. *K*-means clustering (*k* = 7) was applied to capture all possible combinations of interaction patterns between viruses.

### GO enrichment analysis on clusters

Sets of genes found in seven clusters were tested for enrichment of GO terms, which was performed using the enricher function of clusterProfiler package in R (*48*). The GO terms were obtained from the C5 collection of Molecular Signature Database (MSigDBv7.1) and include biological process, cellular component, and molecular function ontologies. Significant GO terms were identified (adjusted *P* < 0.05) and further refined to select nonredundant terms. To select nonredundant gene sets, we first constructed a GO term tree based on distances (1 − Jaccard similarity coefficients of shared genes) between the significant terms. The GO term tree was cut at a specific level (*h* = 0.99) to identify clusters of nonredundant gene sets. For results with multiple significant terms belonging to the same cluster, we selected the term with the lowest adjusted *P* value.

### Sequence similarity analysis

Protein sequence similarity was assessed by comparing the protein sequences from SARS-CoV-1 and MERS-CoV to SARS-CoV-2 for orthologous viral bait proteins. The corresponding PPI similarity was represented by a Jaccard index, using the high-confidence interactomes for each virus.

### GO enrichment and PPI similarity analysis

The high-confidence interactors of the three viruses were tested for enrichment of GO terms as described above. We then identified GO terms that are significantly enriched (adjusted *P* value < 0.05) in all three viruses. For each enriched term, we generated the list of its associated genes and computed the Jaccard index of pairwise comparisons of the three viruses.

### Orthologous versus nonorthologous interactions analysis

For a given pair of viruses, we identified all pairs of baits that share interactors and categorized these into orthologous and nonorthologous groups on the basis of whether the two baits were orthologs or not. We then summed up the total number of shared interactors in each group to calculate the corresponding fractions. This was performed for all pairwise combinations of the three viruses.

### Structural modeling and comparison of MERS-CoV ORF4a and SARS-CoV-2 Nsp8

To obtain a sensitive sequence comparison between MERS-CoV ORF4a and SARS-CoV-2 Nsp8, we took into consideration their homologs. We first searched for homologs of these proteins in the UniRef30 database using hhblits (1 iteration, E-value cutoff 1 × 10^−3^) (*49*). Subsequently, the resulting alignments were filtered to include only sequences with at least 80% coverage to the corresponding query sequence, and hidden Markov models (HMMs) were created using hhmake. Finally, the HMMs of ORF4a and Nsp8 homologs were locally aligned using hhalign. The structure of ORF4a was predicted de novo using trRosetta (*50*). To provide greater coverage than that provided by experimental structures, SARS-CoV-2 Nsp8 was modeled using the structure of its SARS-CoV homolog as template (PDB ID: 2AHM) (*51*) using SWISS-MODEL (*52*). To search for local structural similarities between ORF4a and Nsp8, we used Geometricus, a structure embedding tool based on three-dimensional (3D) rotation invariant moments (*53*). This generates so-called shape-mers, analogous to sequence k-mers. The structures were fragmented into overlapping k-mers on the basis of the sequence (*k* = 20) and into overlapping spheres surrounding each residue (radius = 15 Å). To ensure that the similarities found between these distinct structures were significant, we used a high resolution of 7 to define the shape-mers. This resulted in the identification of four different shape-mers common to ORF4a and Nsp8. We aligned the entire ORF4a structure with residues 96 to 191 of the Nsp8 structure (i.e., after removal of the long N-terminal helix) using the Caretta structural alignment algorithm (*54*), using 3D rotation invariant moments (*53*) for initial superposition. We optimized parameters to maximize the Caretta score. The resulting alignment used *k* = 30, radius = 16 Å, gap open penalty = 0.05, gap extend penalty = 0.005, and had a root mean square deviation (RMSD) of 7.6 Å across 66 aligning residues.

### DIS analysis

We computed a DIS for interactions that (i) originated from viral bait proteins shared across all three viruses and (ii) passed the high-confidence scoring criteria (see above) in at least one virus. We defined the DIS to be the difference between the interaction scores (*K*) from each virus. A DIS near 0 indicates that the interaction is confidently shared between the two viruses being compared, whereas a DIS near −1 or +1 indicates that the host-protein interaction is specific for one virus or the other. We computed a fourth DIS (SARS-MERS) by averaging *K* from SARS-CoV-1 and SARS-CoV-2 before calculating the difference with MERS-CoV. Here, a DIS near +1 indicates SARS-specific interactions (shared between SARS-CoV-1 and SARS-CoV-2 but absent in MERS-CoV), a DIS near −1 indicates MERS-specific interactions (present in MERS-CoV and absent or lowly confident in both SARS-CoVs), and a DIS near 0 indicates interactions shared between all three viruses.

For each pairwise virus comparison, as well as the SARS-MERS comparison, the DIS was defined on the basis of cluster membership of interactions (Fig. 3A). For the SARS2-SARS1 comparison, interactions from every cluster except 5 were used, as those interactions are considered absent from both SARS-CoV-2 and SARS-CoV-1. For the SARS2-MERS comparison, interactions from all clusters except 3 were used. For the SARS1-MERS comparison, interactions from all clusters except 6 were used. For the SARS-MERS comparison, only interactions from clusters 2, 4, and 5 were used.

### Network generation and visualization

PPI networks were generated in Cytoscape (*55*) and subsequently annotated using Adobe Illustrator. Host-host physical interactions, protein complex definitions, and biological process groupings were derived from CORUM (*46*), GO (biological process), and manually curated from literature sources. All networks were deposited in NDEx (*56*).

### siRNA library and transfection in A549-ACE2 cells

An OnTargetPlus siRNA SMARTpool library (Horizon Discovery) was purchased targeting 331 of the 332 human proteins previously identified to bind SARS-CoV-2 (*5*) (PDE4DIP was not available for purchase and was excluded from the assay). This library was arrayed in a 96-well format, with each plate also including two nontargeting siRNAs and one siRNA pool targeting ACE2 (table S12). The siRNA library was transfected into A549 cells stably expressing ACE2 (A549-ACE2, provided by O. Schwartz), using Lipofectamine RNAiMAX reagent (Thermo Fisher). Briefly, 6 pmol of each siRNA pool were mixed with 0.25 μl RNAiMAX transfection reagent and OptiMEM (Thermo Fisher) in a total volume of 20 μl. After a 5 min incubation period, the transfection mix was added to cells seeded in a 96-well format. Twenty-four hours after transfection, the cells were subjected to SARS-CoV-2 infection, as described in the section Viral infection and quantification assay in A549-ACE2 cells, or incubated for 72 hours to assess cell viability using the CellTiter-Glo luminescent viability assay according to the manufacturer’s protocol (Promega). Luminescence was measured in a Tecan Infinity 2000 plate reader, and percentage viability calculated relative to untreated cells (100% viability) and cells lysed with 20% ethanol or 4% formalin (0% viability), included in each experiment.

### Viral infection and quantification assay in A549-ACE2 cells

Cells seeded in a 96-well format were inoculated with a SARS-CoV-2 stock (BetaCoV/France/IDF0372/2020 strain, generated and propagated once in Vero E6 cells and a gift from the National Reference Centre for Respiratory Viruses at Institut Pasteur, Paris, originally supplied through the European Virus Archive goes Global platform) at a MOI of 0.1 PFU per cell. After a 1-hour incubation period at 37°C, the virus inoculum was removed, and replaced by DMEM containing 2% FBS (Gibco, Thermo Fisher). Seventy-two hours postinfection, the cell culture supernatant was collected, heat inactivated at 95°C for 5 min, and used for RT-qPCR analysis to quantify viral genomes present in the supernatant. Briefly, SARS-CoV-2–specific primers targeting the N gene region: 5′-TAATCAGACAAGGAACTGATTA-3′ (forward) and 5′-CGAAGGTGTGACTTCCATG-3′ (reverse) (*57*) were used with the Luna Universal One-Step RT-qPCR Kit (New England Biolabs) in an Applied Biosystems QuantStudio 6 thermocycler, with the following cycling conditions: 55°C for 10 min, 95°C for 1 min, and 40 cycles of 95°C for 10 s, followed by 60°C for 1 min. The number of viral genomes is expressed as PFU equivalents per milliliter, and was calculated by performing a standard curve with RNA derived from a viral stock with a known viral titer.

### Knockdown validation with RT-qPCR in A549-ACE2 cells

Gene-specific qPCR primers targeting all genes represented in the OnTargetPlus library were purchased and arrayed in a 96-well format identical to that of the siRNA library (IDT; table S13). A549-ACE2 cells treated with siRNA were lysed using the Luna Cell Ready Lysis Module (New England Biolabs) following the manufacturer’s protocol. The lysate was used directly for gene quantification by RT-qPCR with the Luna Universal One-Step RT-qPCR Kit (New England Biolabs), using the gene-specific PCR primers and glyceraldehyde-3-phosphate dehydrogenase (GAPDH) as a housekeeping gene. The following cycling conditions were used in an Applied Biosystems QuantStudio 6 thermocycler: 55°C for 10 min, 95°C for 1 min, and 40 cycles of 95°C for 10 s, followed by 60°C for 1 min. The fold change in gene expression for each gene was derived using the 2^−ΔΔCT^, 2 (Delta Delta CT) method (*58*), normalized to the constitutively expressed housekeeping gene *GAPDH*. Relative changes were generated comparing the control siRNA knockdown transfected cells to the cells transfected with each siRNA.

### Single guide RNA selection for Cas9 knockout screen

Single guide RNAs (sgRNAs) were designed according to Synthego’s multiguide gene knockout (*59*). Briefly, two or three sgRNAs are bioinformatically designed to work in a cooperative manner to generate small, knockout-causing, fragment deletions in early exons (fig. S18). These fragment deletions are larger than standard indels generated from single guides. The genomic repair patterns from a multiguide approach are highly predictable on the basis of the guide spacing and design constraints to limit off-targets, resulting in a higher probability protein knockout phenotype (table S14).

### sgRNA synthesis for Cas9 knockout screen

RNA oligonucleotides were chemically synthesized on Synthego solid-phase synthesis platform, using CPG solid support containing a universal linker. 5-benzylthio-1H-tetrazole (BTT, 0.25 M solution in acetonitrile) was used for coupling, [3-((dimethylamino-methylidene)amino)-3H-1,2,4-dithiazole-3-thione (DDTT, 0.1 M solution in pyridine)] was used for thiolation, dichloroacetic acid (DCA, 3% solution in toluene) was used for detritylation. Modified sgRNA were chemically synthesized to contain 2′-O-methyl analogs and 3′ phosphorothioate nucleotide interlinkages in the terminal three nucleotides at both 5′ and 3′ ends of the RNA molecule. After synthesis, oligonucleotides were subject to a series of deprotection steps, followed by purification by solid-phase extraction (SPE). Purified oligonucleotides were analyzed by electrospray ionization mass spectrometry (ESI-MS).

### Arrayed knockout generation with Cas9-RNPs

For Caco-2 transfection, 10 pmol Streptococcus Pyogenes NLS-Sp.Cas9-NLS (SpCas9) nuclease (Aldevron; 9212) was combined with 30 pmol total synthetic sgRNA (10 pmol each sgRNA, Synthego) to form ribonucleoproteins (RNPs) in 20 μl total volume with SF Buffer (Lonza V5SC-2002) and allowed to complex at room temperature for 10 min.

All cells were dissociated into single cells using TrypLE Express (Gibco), resuspended in culture media and counted. 100,000 cells per nucleofection reaction were pelleted by centrifugation at 200 × *g* for 5 min. After centrifugation, cells were resuspended in transfection buffer according to cell type and diluted to 2 × 10^4^ cells/μl. Five μl of cell solution was added to preformed RNP solution and gently mixed. Nucleofections were performed on a Lonza HT 384-well nucleofector system (Lonza, #AAU-1001) using program CM-150 for Caco-2. Immediately after nucleofection, each reaction was transferred to a tissue-culture treated 96-well plate containing 100 μl of normal culture media and seeded at a density of 50,000 cells per well. Transfected cells were incubated following standard protocols.

### Quantification of arrayed knockout efficiency

Two days after nucleofection, genomic DNA was extracted from cells using DNA QuickExtract (Lucigen, #QE09050). Briefly, cells were lysed by removal of the spent media followed by addition of 40 μl of QuickExtract solution to each well. Once the QuickExtract DNA Extraction Solution was added, the cells were scraped off the plate into the buffer. After transfer to compatible plates, DNA extract was then incubated at 68°C for 15 min followed by 95°C for 10 min in a thermocycler before being stored for downstream analysis.

Amplicons for indel analysis were generated by PCR amplification with NEBNext polymerase (NEB, #M0541) or AmpliTaq Gold 360 polymerase (Thermo Fisher Scientific, #4398881) according to the manufacturer’s protocol. The primers were designed to create amplicons between 400 and 800 base pairs (bp), with both primers at least 100 bp from any of the sgRNA target sites (table S15). PCR products were cleaned-up and analyzed by Sanger sequencing (Genewiz). Sanger data files and sgRNA target sequences were input into Inference of CRISPR Edits (ICE) analysis (ice.synthego.com) to determine editing efficiency and to quantify generated indels (*60*). Percentage of alleles edited is expressed as an ice-d score. This score is a measure of how discordant the sanger trace is before versus after the edit. It is a simple and robust estimate of editing efficiency in a pool, especially suited to highly disruptive editing techniques like multiguide.

### Identification of essential genes for siRNA and Cas9 knockout screen

We used longitudinal imaging in A549 cells to assess cell viability (fig. S18). For benchmarking, relative cell viability was measured by CellTiter-Glo Luminescent Cell Viability Assay (Promega; G7571) as per manufacturer’s instructions. Briefly, two passages postnucleofection A549 siRNA pools cultured in 96-well tissue-culture treated plates (Corning, #3595) were lysed in the CellTIter-Glo reagent, by removing spent media and adding 100 μl of the CellTiter-Glo reagent containing the CellTiter-Glo buffer and CellTiter-Glo Substrate. Cells were placed on an orbital shaker for 2 min on a SpectraMax iD5 (Molecular Devices) and then incubated in the dark at room temperature for 10 min. Completely lysed cells were pipette mixed and 25 μl were transferred to a 384-well assay plate (Corning, #3542). The luminescence was recorded on a SpectraMax iD5 (Molecular Devices) with an integration time of 0.25 s per well. Luminescence readings were all normalized to the without-sgRNA control condition.

To determine cell viability in Caco-2 knockouts we used longitudinal imaging (fig. S18). All gene knockout pools were maintained for a minimum of six passages to determine the effect of loss of protein function on cell fitness before viral infection. Viability was determined through longitudinal imaging and automated image analysis using a Celigo Imaging Cytometer (Celigo). Each gene knockout pool was split in triplicate wells on separate plates. Every day, except the day of seeding, each well was scanned and analyzed using built-in Confluence imaging parameters using autoexposure and autofocus with an offset of −45 μm. Analysis was performed with standard settings except for an intensity threshold setting of 8. Confluency was averaged across three wells and plotted over time. Viability genes were determined as pools that, after six passages, remained <20% confluent 5 days after seeding. Genes deemed essential were excluded from the knockout screen.

### Cells, virus, and infections for Caco-2 Cas9 knockout screen

Wild-type and CRISPR-edited Caco-2 cells were grown at 37°C, 5% CO_2_ in DMEM, 10% FBS. SARS-CoV-2 stocks were grown and titered on Vero E6 cells as described previously (*61*). Wild-type and CRISPR-edited Caco-2 cell lines were infected with SARS-CoV-2 at a MOI of 0.01 in DMEM supplemented with 2% FBS. Seventy-two hours postinfection, supernatants were harvested and stored at −80°C and the Caco-2 wild-type (WT) and CRISPR knockout (KO) cells were fixed with 10% neutral buffered formalin (NBF) for 1 hour at room temperature to enable further analysis.

### Focus-forming assay for Caco-2 Cas9 knockout screen

Vero E6 cells were plated into 96-well plates at confluence (50,000 cells per well) in DMEM supplemented with 10% heat-inactivated FBS (Gibco). Before infection, supernatants from infected Caco-2 WT and CRISPR KO cells were thawed and serially diluted from 10^−1^ to 10^−8^. Growth media was removed from the Vero E6 cells and 40 μl of each virus dilution was plated. After 1 hour of adsorption at 37°C, 5% CO_2_, 40 μl of 2.4% microcrystalline cellulose (MCC) overlay supplemented with DMEM powdered media (Gibco) to a concentration of 1x was added to each well of the 96-well plate to achieve a final MCC overlay concentration of 1.2%. Plates were then incubated at 37°C, 5% CO_2_ for 24 hours. The MCC overlay was gently removed and cells were fixed with 10% NBF for 1 hour at room temperature. After removal of NBF, monolayers were washed with ultrapure water and ice-cold 100% methanol/0.3% H_2_O_2_ was added for 30 min to permeabilize the cells and quench endogenous peroxidase activity. Monolayers were then blocked for 1 hour in PBS with 5% nonfat dry milk (NFDM). After blocking, monolayers were incubated with SARS-CoV N primary antibody (Novus Biologicals; NB100-56576; 1:2000) for 1 hour at room temperature in PBS, 5% NFDM. Monolayers were washed with PBS and incubated with an HRP-conjugated secondary antibody for 1 hour at room temperature in PBS with 5% NFDM. Secondary antibody was removed, monolayers were washed with PBS, and then developed using TrueBlue substrate (KPL) for 30 min. Plates were imaged on a Bio-Rad Chemidoc utilizing a phosphorscreen and foci were counted by eye to calculate focus-forming units per ml (FFU/ml) for each knockout. The original formalin-fixed Caco-2 WT and CRISPR KO cells were stained with DAPI (Thermo Scientific) and imaged on a Cytation 5-plate reader to determine cell viability. Wells containing no cells were excluded from further analyses.

### Quantitative analysis and scoring of knockdown and knockout library screens

Virus readout by qPCR (A549-ACE2, expressed as plaque-forming units per milliliter) and focus-forming assay readouts (Caco-2, focus-forming units per milliliter) were processed using the RNAither package (www.bioconductor.org/packages/release/bioc/html/RNAither.html) in the statistical computing environment R. The two datasets were normalized separately, using the following method. The readouts were first log transformed (natural logarithm), and robust *z*-scores [using median and MAD (median absolute deviation) instead of mean and standard deviation] were then calculated for each 96-well plate separately. *z*-scores of multiple replicates of the same perturbation were averaged into a final *z*-score for presentation in Fig. 5. No filtering was done on the basis of differences in replicate *z*-scores, but all replicate scores are individually listed in tables S6 and S7. We suggest consulting the replicate *z*-scores for all genes and perturbations of interest. The A549-ACE2 siRNA screen includes three replicates (or more) of each perturbation, and the Caco-2 CRISPR screen includes two replicates (or more) of each perturbation. The results from the A549-ACE2 screen cover all 332 screened genes (331 SARS-CoV-2 interactors plus ACE2). The results from the Caco-2 screen cover 286 of the screened genes plus ACE2. The remaining Caco-2 genes were either deemed essential, failed editing, or failed in the focus-forming assay.

### Antiviral drug and cytotoxicity assays (A549-ACE2 cells)

In total, 2500 A549-ACE2 cells were seeded into 96- or 384-well plates in DMEM (10% FBS) and incubated for 24 hours at 37°C, 5% CO_2_. Two hours before infection, the media was replaced with 120 μl (96-well format) or 50 μl (384-well format) of DMEM (2% FBS) containing the compound of interest at the indicated concentration. At the time of infection, the media was replaced with virus inoculum (MOI 0.1 PFU per cell) and incubated for 1 hour at 37°C, 5% CO_2_. After the adsorption period, the inoculum was removed, replaced with 120 μl (96-well format) or 50 μl (384-well format) of drug-containing media, and cells were incubated for an additional 72 hours at 37°C, 5% CO_2_. At this point, the cell culture supernatant was harvested, and viral load was assessed by RT-qPCR (as described in the section Viral infection and quantification assay in A549-ACE2 cells). Viability was assayed using the CellTiter-Glo assay following the manufacturer’s protocol (Promega). Luminescence was measured in a Tecan Infinity 2000 plate reader, and percentage viability calculated relative to untreated cells (100% viability) and cells lysed with 20% ethanol or 4% formalin (0% viability), included in each experiment.

### Antiviral drug and cytotoxicity assays (Vero E6 cells)

Viral growth and cytotoxicity assays in the presence of inhibitors were performed as previously described (*5*). In total, 2000 Vero E6 cells were seeded into 96-well plates in DMEM (10% FBS) and incubated for 24 hours at 37°C, 5% CO_2_. Two hours before infection, the medium was replaced with 100 μl of DMEM (2% FBS) containing the compound of interest at concentrations 50% greater than those indicated, including a DMSO control. SARS-CoV-2 virus (100 PFU; MOI 0.025) was added in 50 μl of DMEM (2% FBS), bringing the final compound concentration to those indicated. Plates were then incubated for 48 hours at 37°C. After infection, supernatants were removed, and cells were fixed with 4% formaldehyde for 24 hours before being removed from the BSL3 facility. The cells were then immunostained for the viral NP protein (rabbit antisera produced in the Garcia-Sastre laboratory; 1:10,000) with a DAPI counterstain. Infected cells (488 nm) and total cells (DAPI) were quantified using a Celigo (Nexcelcom) imaging cytometer. Infectivity is measured by the accumulation of viral NP protein in the nucleus of the cells (fluorescence accumulation). Percent infection was quantified as {[(number of infected cells / total cells) − background] × 100}, and the DMSO control was then set to 100% infection for analysis. The IC_50_ and IC_90_ for each experiment was determined using the Prism (GraphPad Software) software. Cytotoxicity measurements were performed using the MTT assay (Roche), according to the manufacturer’s instructions. Cytotoxicity was performed in uninfected Vero E6 cells with same compound dilutions and concurrent with viral replication assay. All assays were performed in biologically independent triplicates. Sourcing information for all drugs tested may be found in table S10.

### Coimmunoprecipitation assays for ORF9b and Tom70

HEK293T and A549 cells were transfected with the indicated mammalian expression plasmids using Lipofectamine 2000 (Invitrogen) and TransIT-X2 (Mirus Bio), respectively. Twenty-four hours after transfection, cells were harvested and lysed in NP-40 lysis buffer [0.5% Nonidet P 40 Substitute (NP-40; Fluka Analytical), 50 mM Tris-HCl, pH 7.4 at 4°C, 150 mM NaCl, 1 mM EDTA] supplemented with cOmplete mini EDTA-free protease and PhosSTOP phosphatase inhibitor cocktails (Roche). Clarified cell lysates were incubated with Streptactin Sepharose beads (IBA) for 2 hours at 4°C, followed by five washes with NP-40 lysis buffer. Protein complexes were eluted in the SDS loading buffer and were analyzed by Western blotting with the indicated antibodies.

### Quantification of Tom70 down-regulation in HeLaM cells overexpressing ORF9b

HeLaM cells were transiently transfected with plasmids encoding GFP-Strep, SARS-CoV-1 ORF9b-Strep, or SARS-CoV-2 ORF9b-Strep. The next day, the cells were fixed using 4% paraformaldehyde and immunostained with antibodies against Strep tag, and Tom20 or Tom70. Representative images for each construct were captured by acquiring a single optical section using a Nikon A1 confocal fitted with a CFI Plan Apochromat VC 60x oil objective (NA 1.4). For image quantification multiple fields of view were captured for each construct using a CFI Super Plan Fluor ELWD 40x objective (NA 0.6). The mean fluorescent intensity for Tom20 and Tom70 was measured by manually drawing a region of interest around each cell using ImageJ. Between 30 and 60 cells were quantified for each construct.

### Quantification of Tom70 down-regulation in infected Caco-2 cells

Caco-2 cells were seeded on glass coverslips in triplicate and infected with SARS-CoV-2 at a MOI of 0.1 as described above. At 24 hours postinfection, cells were fixed with 4% paraformaldehyde and immunostained with antibodies against Tom70, Tom20, and ORF9b. For signal quantification images of noninfected and neighboring infected cells were acquired using a LSM800 confocal laser-scanning microscope (Zeiss) equipped with a 63X, 1.4 NA oil objective and the Zen blue software (Zeiss). The mean fluorescence intensity of each cell was measured by ImageJ software. Forty-three cells were quantified for each condition—infected or noninfected—from three independent experiments.

### Coexpression and purification of ORF9b-Tom70 (residues 109 to end) complexes

SARS-CoV-2 ORF9b and Tom70 (residues 109 to end) were coexpressed using a pET29-b(+) vector backbone where ORF9b was tag-less and Tom70 had an N-terminal 10XHis-tag and SUMO-tag. LOBSTR *E. coli* cells transformed with the above construct were grown at 37°C until they reached an optical density at 600 nm (OD_600_) of 0.8, then expression was induced at 37°C with 1 mM IPTG for 4 hours. Frozen cell pellets were resuspended in 25 ml of lysis buffer (200 mM NaCl, 50 mM Tris-HCl pH 8.0, 10% v/v glycerol, 2 mM MgCl_2_) per liter of cell culture, supplemented with cOmplete protease inhibitor tablets (Roche), 1 mM phenylmethylsulfonyl fluoride (PMSF) (Sigma), 100 μg/ml lysozyme (Sigma), 5 μg/ml DNaseI (Sigma), and then homogenized with an immersion blender (Cuisinart). Cells were lysed by 3x passage through an Emulsiflex C3 cell disruptor (Avestin) at ~103,000 kPa, and the lysate clarified by ultracentrifugation at 100,000 × *g* for 30 min at 4°C. The supernatant was collected, supplemented with 20 mM imidazole, loaded into a gravity flow column containing Ni-NTA superflow resin (Qiagen), and rocked with the resin at 4°C for 1 hour. After allowing the column to drain, resin was rinsed twice with 5 column volumes (cv) of wash buffer [150 mM KCl, 30 mM Tris-HCl pH 8.0, 10% v/v glycerol, 20 mM imidazole, 0.5 mM tris(hydroxypropyl)phosphine (THP, VWR)] supplemented with 2 mM ATP (Sigma) and 4 mM MgCl_2_, then washed with 5 cv wash buffer with 40 mM imidazole. Resin was then rinsed with 5 cv Buffer A (50 mM KCl, 30 mM Tris-HCl pH 8.0, 5% glycerol, 0.5 mM THP) and protein was eluted with 2 × 2.5 cv Buffer A plus 300 mM imidazole. Elution fractions were combined, supplemented with Ulp1 protease, and rocked at 4°C for 2 hours. Ulp1-digested Ni-NTA eluate was diluted 1:1 with additional Buffer A, loaded into a 50 ml Superloop, and applied to a MonoQ 10/100 column on an Äkta pure system (GE Healthcare) using 100% Buffer A, 0% Buffer B (1000 mM KCl, 30 mM Tris-HCl pH 8.0, 5% glycerol, 0.5 mM THP). The MonoQ column was washed with 0 to 40% Buffer B gradient over 15 cv, peak fractions were analyzed by SDS-PAGE and the identity of tagless Tom70 (109 to end) and ORF9b proteins confirmed by intact protein MS (Xevo G2-XS Mass Spectrometer, Waters). Peak fractions eluting at ~15% B contained relatively pure Tom70 (109 to end) and ORF9b, and these were concentrated using 10 kDa Amicon centrifugal filter (Millipore) and further purified by size exclusion chromatography using a Superdex 200 increase 10/300 GL column (GE Healthcare) in buffer containing 150 mM KCl, 20 mM HEPES-NaOH pH 7.5, 0.5 mM THP. The sole size-exclusion peak contained both Tom70 (109 to end) and ORF9b, and the center fraction was used directly for cryo-EM grid preparation.

### Expression and purification of SARS-CoV-2 ORF9b

ORF9b with N-terminal 10XHis-tag and SUMO-tag was expressed using a pET-29b(+) vector backbone. LOBSTR *E. coli* cells transformed with the above construct were grown at 37°C until they reached an optical density at 600 nm (OD_600_) of 0.8, then expression was induced at 37°C with 1 mM IPTG for 6 hours. Frozen cell pellets were lysed, homogenized, clarified, and subject to Ni affinity purification as described above for ORF9b-Tom70 complexes, with several small changes. Lysis buffers and Ni-NTA wash buffers contained 500 mM NaCl, and an additional wash step using 10 cv wash buffer plus 0.2% TWEEN20 plus 500 mM NaCl was carried out before the ATP wash. ORF9b was eluted from Ni-NTA resin in Buffer A (50 mM NaCl, 25 mM Tris pH 8.5, 5% glycerol, 0.5 mM THP) supplemented with 300 mM imidazole. This eluate was diluted 1:1 with additional Buffer A, loaded into a 50 ml Superloop, and applied to a MonoQ 10/100 column on an Äkta pure system (GE Healthcare) using 100% Buffer A, 0% Buffer B (1000 mM NaCl, 25mM Tris-HCl pH 8.5, 5% glycerol, 0.5 mM THP). The MonoQ column was washed with 0 to 40% Buffer B gradient over 15 cv, and relatively pure ORF9b eluted at 20 to 25% Buffer B, whereas ORF9b and contaminating proteins eluted at 30 to 35% buffer B. Fractions from these two peaks were combined and incubated with Ulp1 and HRV3C proteases at 4°C for 2 hours, supplemented with 10 mM imidazole, then thrice flowed back through 1 ml of Ni-NTA resin equilibrated with size-exclusion buffer (as above) plus10 mM imidazole. The reverse-Ni purified sample was concentrated using 10 kDa Amicon centrifugal filter and then further purified by size exclusion chromatography using a Superdex 200 increase 10/300 GL column.

### Expression and purification of Tom70 (109-end)

Tom70 (109 to end) with N-terminal 10XHis-tag and SUMO-tag and C terminus Spy-tag, HRV-3C protease cleavage site, and eGFP-tag was expressed using a pET-21(+) vector backbone. LOBSTR *E. coli* cells transformed with the above construct were grown at 37°C until they reached an optical density at 600 nm (OD_600_) of 0.8, then expression was induced at 16°C with 0.5 mM IPTG overnight. The soluble domain of Tom70 [Tom70 (109-end)] was purified as described in (*62*) with some modifications. Frozen cell pellets of LOBSTR *E. coli* transformed with the above construct were resuspended in 50 ml lysis buffer (500 mM NaCl, 20 mM KH*_2_*PO_4_ pH 7.5) per liter cell culture, supplemented with 1 mM PMSF (Sigma) and 100 μg/ml, and homogenized. Cells were lysed by 3x passage through an Emulsiflex C3 cell disruptor (Avestin) at ~103,000 kPa, and the lysate clarified by ultracentrifugation at 100,000 × *g* for 30 min at 4°C. The supernatant was collected, supplemented with 20 mM imidazole, loaded into a gravity flow column containing Ni-NTA superflow resin (Qiagen), and rocked with the resin at 4°C for 1 hour. After allowing the column to drain, resin was rinsed twice with 5 column volumes (cv) of wash buffer (500 mM KCl, 20 mM KH*_2_*PO_4_ pH 8.0, 20 mM imidazole, 0.5 mM THP) supplemented with 2 mM ATP (Sigma) and 4 mM MgCl_2_, then washed with 5 cv wash buffer with 40 mM imidazole. Bound Tom70 (109 to end) was then cleaved from the resin by 2-hour incubation with Ulp1 protease in 4 cv elution buffer (150 mM KCl, 20 mM KH*_2_*PO_4_ pH 8.0, 5 mM imidazole, 0.5 mM THP). After cleavage with Ulp1, the flow through was collected along with a 2-cv rinse of the resin with additional elution buffer. These fractions were combined and HRV3C protease was added to remove the C-terminal EGFP tag (1:20 HRV3C to Tom70). After 2-hour HRV3C digestion at 4°C, the double-digested Tom70 (109 to end) was concentrated using a 30 kDa Amicon centrifugal filter (Millipore) and further purified by size exclusion chromatography using a Superdex 200 increase 10/300 GL column (GE Healthcare) in buffer containing 150 mM KCl, 20 mM HEPES-NaOH pH 7.5, 0.5 mM THP.

### Prediction of SARS-CoV-2 ORF9b internal mitochondrial targeting sequence

ORF9b was analyzed for the presence of an internal mitochondrial targeting sequence (i-MTS) as described in (*63*) using the TargetP-2.0 server (*64*). Sequences corresponding to ORF9b N-terminal truncations of 0 to 62 residues were submitted to the TargetP-2.0 server, and the probability of the peptides containing an MTS plotted against the numbers of residues truncated. A similar analysis using the MitoFates server (*65*) predicted that ORF9b residues 54 to 63 were the most likely to make up a presequence MTS on the basis of their propensity to form a positively charged amphipathic helix. Notably this analysis was consistent with the secondary structure prediction from JPRED (*66*).

### Cryo-EM sample preparation and data collection

Three μl of ORF9b-Tom70 complex (12.5 μM) was added to a 400 mesh 1.2/1.3R Au Quantifoil grid previously glow discharged at 15 mA for 30 s. Blotting was performed with a blot force of 0 for 5 s at 4°C and 100% humidity in a FEI Vitrobot Mark IV (ThermoFisher) before plunge freezing into liquid ethane. A total of 1534 118-frame super-resolution movies were collected with a 3 by 3 image-shift collection strategy at a nominal magnification of 105,000x (physical pixel size: 0.834 Å per pixel) on a Titan Krios (ThermoFisher) equipped with a K3 camera and a Bioquantum energy filter (Gatan) set to a slit width of 20 eV. Collection dose rate was 8 electrons per pixel per second for a total dose of 66 electrons per square angstrom. Defocus range was −0.7 to −2.4 μm. Each collection was performed with semiautomated scripts in SerialEM (*67*).

### Cryo-EM image processing and model building

We motion corrected 1534 movies using Motioncor2 (*68*), and we imported dose-weighted summed micrographs in cryosparc (version 2.15.0). Then, 1427 micrographs were curated on the basis of contrast transfer function (CTF) fit (better than 5 Å) from a patch CTF job. Template-based particle picking resulted in 2,805,121 particles, and 1,616,691 particles were selected after 2D classification. Five rounds of 3D classification using multiclass ab initio reconstruction and heterogeneous refinement yielded 178,373 particles. Homogeneous refinement of these final particles led to a 3.1-Å electron density map which was used for model building. The reconstruction was filtered by the masked Fourier shell correlation (FSC) and sharpened with a b-factor of −145.

To build the model of Tom70 (109 to end), the crystal structure of *Saccharomyces cerevisiae* Tom71 (PDB ID: 3fp3; sequence identity 25.7%) was first fit into the cryo-EM density as a rigid body in UCSF ChimeraX and then relaxed into the final density using Rosetta FastRelax mover in torsion space. This model, along with a BLAST alignment of the two sequences (*69*), was used as a starting point for manual building using COOT (*70*). After initial building by hand, the regions with poor density fit or geometry were iteratively rebuilt using Rosetta (*71*). ORF9b was built de novo into the final density using COOT, informed and facilitated by the predictions of the TargetP-2.0, MitoFates, and JPRED servers. The ORF9b-Tom70 complex model was submitted to the Namdinator web server (*72*) and further refined in ISOLDE 1.0 (*73*) using the plugin for UCSF ChimeraX (*74*). Final model b-factors were estimated using Rosetta. The model was validated using phenix.validation_cryoem (*75*). The final model contains residues 109 to 272 and 298 to 600 of human Tom70 and 39 to 76 of SARS-CoV-2 ORF9b. Molecular interface between ORF9b and Tom70 was analyzed using the PISA web server (*76*). Figures were prepared using UCSF ChimeraX.

### Computational human genetics analysis

To look for genetic variants associated with our list of proteins that had a meaningful impact on SARS-CoV-2 replication, we used the largest proteomic GWAS study to date (*28*). We identified IL17RA as one of the proteins assayed in Sun *et al*.’s proteomic GWAS and observed that it had multiple cis-acting protein quantitative trait loci (pQTLs) at a corrected *P* value of 1 × 10^−5^, where cis-acting is defined as within 1 Mb of the transcription start site of *IL17RA*.

We used the GSMR method (*29*) to perform Mendelian randomization (MR) using near-independent [linkage disequilibrium (LD) *R*^2^ = 0.05, where *R*^2^ is the coefficient of determination] cis-pQTLs for *IL17RA*. The advantage of the GSMR method over conventional MR methods is twofold. First, GSMR performs MR adjusting for any residual correlation between selected genetic variants by default. Second, GSMR has a built-in method called HEIDI (heterogeneity in dependent instruments)–outlier that performs heterogeneity tests in the near-independent genetic instruments and removes potentially pleiotropic instruments (i.e., where there is evidence of heterogeneity at *P* < 0.01). Details of the GSMR and HEIDI method have been published previously (*29*).

Summary statistics generated by COVID-HGI (round 3; www.covid19hg.org/results/) for COVID-19 versus population, hospitalized COVID-19 versus population and hospitalized COVID-19 versus nonhospitalized COVID-19 were used for *IL17RA* MR analysis. We used the 1000 genomes phase 3 European population genotype data to derive the LD correlation matrix for this analysis. The phenotype definitions as provided by COVID-HGI are as follows. COVID-19 versus population: Case, individuals with laboratory confirmation of SARS-CoV-2 infection, EHR/ICD coding/Physician-confirmed COVID-19, or self-reported COVID-19 positive; control, everybody that is not a case. Hospitalized COVID-19 versus population: case, hospitalized, laboratory confirmed SARS-CoV-2 infection or hospitalization due to COVID-19-related symptoms; control, everybody that is not a case, e.g., population. Hospitalized COVID-19 versus nonhospitalized COVID-19: case, hospitalized, laboratory confirmed SARS-CoV-2 infection or hospitalization due to COVID-19-related symptoms; control, laboratory confirmed SARS-CoV-2 infection and not hospitalized 21 days after the test.

### Infections and treatments for IL-17A treatment studies

The WA-1 strain (BEI resources) of SARS-CoV-2 was used for all experiments. All live virus experiments were performed in a BSL3 laboratory. SARS-CoV-2 stocks were passaged in Vero E6 cells (ATCC) and titer was determined via plaque assay on Vero E6 cells as previously described (*77*). Briefly, virus was diluted 1:10^2^ to 1:10^6^ and incubated for 1 hour on Vero E6 cells before an overlay of Avicel and complete DMEM (Sigma Aldrich, SLM-241) was added. After incubation at 37°C for 72 hours, the overlay was removed and cells were fixed with 10% formalin, stained with crystal violet, and counted for plaque formation. SARS-CoV-2 infections of A549-ACE2 cells were done at a MOI of 0.05 for 24 hours. Inhibitors and cytokines were added concurrently with virus. All infections were done in technical triplicate. Cells were treated with the following compounds: Remdesivir (SELLECK CHEMICALS LLC, S8932) and IL-17A (Millipore-Sigma, SRP0675).

### RNA extraction, RT, and RT-qPCR for IL-17A treatment studies

Total RNA from samples was extracted using the Direct-zol RNA kit (Zymogen, R2060) and quantified using the NanoDrop 2000c (ThermoFisher). cDNA was generated using 500 ng of RNA from infected A549-ACE2 cells with Superscript III reverse transcription (ThermoFisher, 18080-044) and oligo(dT)_12-18_ (ThermoFisher, 18418-012) and random hexamer primers (ThermoFisher, S0142). RT-qPCR reactions were performed on a CFX384 (BioRad) and delta cycle threshold (ΔCt) was determined relative to RPL13A levels. Viral detection levels and target host genes in treated samples were normalized to water-treated controls. The SYBR green qPCR reactions contained 5 μl of 2x Maxima SYBR green/Rox qPCR Master Mix (ThermoFisher; K0221), 2 μl of diluted cDNA, and 1 nmol of both forward and reverse primers, in a total volume of 10 μl. The reactions were run as follows: 50°C for 2 min and 95°C for 10 min, followed by 40 cycles of 95°C for 5 s and 62°C for 30 s. Primer efficiencies were ~100%. Dissociation curve analysis after the end of the PCR confirmed the presence of a single and specific product. RT-qPCR primers were used against the SARS-CoV-2 E gene (PF_042_nCoV_E_F: ACAGGTACGTTAATAGTTAATAGCGT; PF_042_nCoV_E_R: ATATTGCAGCAGTACGCACACA), the CXCL8 gene (CXCL8 For: ACTGAGAGTGATTGAGAGTGGAC; CXCL8 Rev: AACCCTCTGCACCCAGTTTTC), and the RPL13A gene (RPL13A For: CCTGGAGGAGAAGAGGAAAGAGA; RPL13A Rev: TTGAGGACCTCTGTGTATTTGTCAA).

### Transfections for IL-17A treatment studies

HEK293T cells were seeded 5 × 10^5^cells per well (in 6-well plate) or 3 × 10^6^ cells per 10-cm^2^ plates. The next day, 2 or 10 μg of plasmids was transfected using X-tremeGENE 9 DNA Transfection Reagent (Roche) in 6-well plate or 10-cm^2^ plates, respectively. For IL-17A (Millipore-Sigma, SRP0675) incubation in cells, 0.5 μg of IL-17A was treated either before or after transfection and incubated at 37°C. After 48 hours, cells were collected by trypsinization. For IL-17A incubation with cell lysates, transfected cell lysates were incubated in the presence of 0.5 or 5 μg/ml IL-17A at 4°C on a rotator overnight. Plasmids pLVX-EF1alpha–SARS-CoV-2–orf8-2xStrep-IRES-Puro (ORF8) and pLVX-EF1alpha-eGFP-2xStrep-IRES-Puro (EGFP-Strep) were a gift from N. J. Krogan. (Addgene plasmid #141390, 141395) (*5*). pLVX-EF1alpha- IRES-Puro (Vector) was obtained from Takara/Clontech.

### SARS-CoV-2 ORF8 and IL17RA coimmunoprecipitation

Transfected and treated HEK293T cells were pelleted and washed in cold D-PBS and later resuspended in Flag-IP Buffer (50 mM Tris HCl, pH 7.4, with 150 mM NaCl, 1 mM EDTA, and 1% NP-40) with 1x HALT (ThermoFisher Scientific, 78429), incubated with buffer for 15 min on ice then centrifuged at 13,000 rpm for 5 min. The supernatant was collected and 1 mg of protein was used for immunoprecipitation (IP) with 100 μl of Streptactin Sepharose (IBA, 2-1201-010) on a rotor overnight at 4°C. Immunoprecipitates were washed five times with Flag-IP buffer and eluted with 1x Buffer E (100 mM Tris-Cl, 150 mM NaCl, 1 mM EDTA, 2.5 mM Desthiobiotin). Eluate was diluted with 1x-NuPAGE (ThermoFisher Scientific, #NP0008) LDS Sample Buffer with 2.5% β-Mercaptoethanol and blotted for targeted antibodies. Antibodies used were Strep tag II (Qiagen, #34850), B-Actin (Sigma, #A5316), and IL17RA (Cell Signaling, #12661S).

### Computational docking of PGES-2 and Nsp7

A model for human PGES-2 dimer was constructed by homology using MODELER (*78*) from the crystal structure of *Macaca fascularis* mPGES-2 [PDB ID: 1Z9H (*79*); 98% sequence identity] bound to indomethacin. Indomethacin was removed from the structure utilized for docking. The structure of SARS-CoV-2 Nsp7 was extracted from PDB ID 7BV2 (*80*). Docking models were produced using ClusPro (*81*), ZDock (*82*), HDock (*83*), Gramm-X (*84*), SwarmDock (*85*), and PatchDock (*86*) with SOAP-PP score (*87*). For each protocol, up to 100 top scoring models were extracted (fewer for those that do not report >100 models); for PatchDock, models with SOAP-PP *z*-scores >3.0 were used (fig. S23A). The 420 models were clustered at 4.0-Å RMSD, resulting in 127 clusters. The two largest clusters, composed of 192 models, are related by dimer symmetry. All other clusters contain <15 models.

### Assessment of positive selection signatures in SIGMAR1

SIGMAR1 protein alignments were generated from whole-genome sequences of 359 mammals curated by the Zoonomia consortium. Protein alignments were generated with TOGA (https://github.com/hillerlab/TOGA), and missing sequence gaps were refined with CACTUS (*88*, *89*). Branches undergoing positive selection were detected with the branch-site test aBSREL (*90*) implemented in the HyPhy package (*90*, *91*). PhyloP was used to detect codons undergoing accelerated evolution along branches detected as undergoing positive selection by aBSREL relative to the neutral evolution rate in mammals, determined using phyloFit on third nucleotide positions of codons which are assumed to evolve neutrally. *P* values from phyloP were corrected for multiple tests using the Benjamini-Hochberg method (*92*). PhyloFit and phyloP are both part of the PHAST package version 1.4 (*93*, *94*).

### Comparative SARS-CoV-1 inhibition by amiodarone

SARS-CoV-1 (Urbani) drug screens were performed with Vero E6 cells (ATCC #1568, Manassas, VA) cultured in DMEM (Quality Biological), supplemented with 10% (v/v) heat-inactivated FBS (Sigma), 1% (v/v) penicillin-streptomycin (Gemini Bio-products), and 1% (v/v) L-glutamine (2 mM final concentration, Gibco). Cells were plated in opaque 96-well plates 1 day before infection. Drugs were diluted from stock to 50 μM and an 8-point 1:2 dilution series prepared in duplicate in Vero Media. Every compound dilution and control were normalized to contain the same concentration of drug vehicle (e.g., DMSO). Cells were pretreated with drug for 2 hours at 37°C (5% CO2) before infection with SARS-CoV-1 at MOI 0.01. In addition to plates that were infected, parallel plates were left uninfected to monitor cytotoxicity of drug alone. All plates were incubated at 37°C (5% CO2) for 3 days before performing CellTiter-Glo (CTG) assays as per the manufacturer’s instruction (Promega, Madison, WI). Luminescence was read on a BioTek Synergy HTX plate reader (BioTek Instruments Inc., Winooski, VT) using the Gen5 software (version 7.07, Biotek Instruments Inc., Winooski, VT).

### Real-world data source and analysis

This study used deidentified patient-level records from HealthVerity’s Marketplace dataset, a nationally representative dataset covering >300 million patients with medical and pharmacy records from >60 health care data sources in the United States. The current study used data from 738,933 patients with documented COVID-19 infection between 1 March 2020 and 17 August 2020, defined as a positive or presumptive positive viral laboratory test result or an International Classification of Diseases, 10th Revision, Clinical Modification (ICD-10-CM) diagnosis code of U07.1 (COVID-19).

For this population, we analyzed medical claims, pharmacy claims, laboratory data, and hospital chargemaster data containing diagnoses, procedures, medications, and COVID-19 laboratory results from both inpatient and outpatient settings. Claims data included open (unadjudicated) claims sourced in near-real time from practice management and billing systems, claims clearinghouses, and laboratory chains, as well as closed (adjudicated) claims encompassing all major U.S. payer types (commercial, Medicare, and Medicaid). For inpatient treatment evaluations, we used linked hospital chargemaster data containing records of all billable procedures, medical services, and treatments administered in hospital settings. Linkage of patient-level records across these data types provides a longitudinal view of baseline health status, medication use, and COVID-19 progression for each patient under study. Data for this study covered the period of 1 December 2018 through 17 August 2020. All analyses were conducted with the Aetion Evidence Platform version r4.6.

This study was approved by the New England institutional review board (IRB) (no. 1-9757-1). Medical records constitute protected health information and can be made available to qualified individuals on reasonable request.

### Observation of hospitalization outcomes in outpatient new users of indomethacin (treatment arm) versus celecoxib (active comparator) using real-world data

We used an incident (new) user, active comparator design (*95*, *96*) to assess the risk of hospitalization among newly diagnosed COVID-19 patients who were subsequently treated with indomethacin or the comparator agent, celecoxib. Patients were required to have COVID-19 infection recorded in an outpatient setting during the study period of 1 March 2020 to 17 August 2020 and occurring in the 21 days before (and including) the date of indomethacin or celecoxib treatment initiation. Prevalent users of prescription-only NSAIDs (any prescription fill for indomethacin, celecoxib, ketoprofen, meloxicam, sulindac, or piroxicam 60 days prior) and patients hospitalized in the 21 days before and including the date of treatment initiation were excluded from this analysis.

Using RSS, patients treated with indomethacin were matched at a 1:1 ratio to controls randomly selected among patients treated with celecoxib, with direct matching on calendar date of treatment (±7 days), age (±5 years), sex, Charlson comorbidity index (exact) (*97*), time since confirmed COVID-19 (±5 days), and disease severity based on the highest-intensity COVID-19–related health service in the 7 days before and including the date of treatment initiation (laboratory service only versus outpatient medical visit versus emergency department visit) and symptom profile in the 21 days before and including the date of treatment initiation (recorded symptoms versus none). This risk-set–sampled population was further matched on a PS (*33*) estimated using logistic regression with 24 demographic and clinical risk factors, including covariates related to baseline medical history and COVID-19 severity in the 21 days before treatment (table S11). Balance between indomethacin and celecoxib treatment groups was evaluated by comparison of absolute standardized differences in covariates, with an absolute standardized difference of <0.2 indicating good balance between the treatment groups (*98*).

The primary analysis was an intention-to-treat design, with follow-up beginning 1 day after indomethacin or celecoxib initiation and ending on the earliest occurrence of 30 days of follow-up reached or end of patient data. ORs for the primary outcome of all-cause inpatient hospitalization were estimated for the RSS-plus-PS–matched population as well as for the RSS-matched population. Our primary outcome definition required a record of inpatient hospital admission with a resulting inpatient stay; as a sensitivity, a broader outcome definition captured any hospital visit (defined with revenue and place of service codes).

### Observation of mechanical ventilation outcomes in inpatient new users of typical antipsychotics (treatment arm) versus atypical antipsychotics (active comparator) using real-world data

We used an incident user, active comparator design (*95*, *96*) to assess the risk of mechanical ventilation among hospitalized COVID-19 patients treated with typical or atypical antipsychotics in an inpatient setting. See table S11 for a list of drugs included in each category. To permit assessment of day-level in-hospital confounders and outcomes, this analysis was restricted to hospitalized patients observable in hospital chargemaster data. Prevalent users of typical or atypical antipsychotics (any prescription fill or chargemaster-documented use in 60 days prior) and patients with evidence of mechanical ventilation in the 21 days before and including the date of treatment initiation were excluded from this analysis.

Using RSS, hospitalized patients treated with typical antipsychotics were matched at a 1:1 ratio to controls randomly selected among patients treated with atypical antipsychotics, with direct matching (1:1 fixed ratio) on calendar date of treatment (±7 days), age (±5 years), sex, Charlson comorbidity index (exact) (*97*), time since hospital admission, and disease severity as defined with a simplified version of the World Health Organization’s ordinal scale for clinical improvement (*99*). This risk-set–sampled population was further matched on a PS estimated using logistic regression with 36 demographic and clinical risk factors, including covariates related to baseline medical history, admitting status, and disease severity at treatment (table S11). Balance between typical and atypical treatment groups was evaluated by comparison of absolute standardized differences in covariates, with an absolute standardized difference of <0.2 indicating good balance between the treatment groups (*98*).

The primary analysis was an intention-to-treat design, with follow up beginning 1 day after the date of typical or atypical antipsychotic treatment initiation and ending on the earliest occurrence of 30 days of follow-up reached, discharge from hospital, or end of patient data. ORs for the primary outcome of inpatient mechanical ventilation were estimated for the RSS-plus-PS–matched population as well as for the RSS-matched population.

## Notes

**Correction (21 December 2020):** The Acknowledgments section has been updated to include an additional funding source for Melanie Ott and Nevan J. Krogan.

## Supplementary Materials

science.sciencemag.org/content/370/6521/eabe9403/suppl/DC1 QCRG Structural Biology Consortium Author List Zoonomia Consortium Author List Figs. S1 to S25 Tables S1 to S15 Reference (*101*) MDAR Reproducibility Checklist Movie S1

## Appendix

View/request a protocol for this paper from *Bio-protocol*.

## References

[1] J. Liu, W. Xie, Y. Wang, Y. Xiong, S. Chen, J. Han, Q. Wu, A comparative overview of COVID-19, MERS and SARS: Review article. Int. J. Surg. 81, 1–8 (2020). 10.1016/j.ijsu.2020.07.032 32730205 PMC7382925

[2] J. H. Beigel, K. M. Tomashek, L. E. Dodd, A. K. Mehta, B. S. Zingman, A. C. Kalil, E. Hohmann, H. Y. Chu, A. Luetkemeyer, S. Kline, D. Lopez de Castilla, R. W. Finberg, K. Dierberg, V. Tapson, L. Hsieh, T. F. Patterson, R. Paredes, D. A. Sweeney, W. R. Short, G. Touloumi, D. C. Lye, N. Ohmagari, M.-d. Oh, G. M. Ruiz-Palacios, T. Benfield, G. Fätkenheuer, M. G. Kortepeter, R. L. Atmar, C. B. Creech, J. Lundgren, A. G. Babiker, S. Pett, J. D. Neaton, T. H. Burgess, T. Bonnett, M. Green, M. Makowski, A. Osinusi, S. Nayak, H. C. Lane, ACTT-1 Study Group Members, Remdesivir for the treatment of Covid-19—Final report. N. Engl. J. Med. 383, 1813–1826 (2020). 10.1056/NEJMoa2007764 32445440 PMC7262788

[3] RECOVERY Collaborative Group, Dexamethasone in Hospitalized Patients with Covid-19 - Preliminary Report. N. Engl. J. Med. 10.1056/nejmoa2021436 (2020). 10.1056/nejmoa2021436 32678530 PMC7383595

[4] M. Becerra-Flores, T. Cardozo, SARS-CoV-2 viral spike G614 mutation exhibits higher case fatality rate. Int. J. Clin. Pract. 74, e13525 (2020). 10.1111/ijcp.13525 32374903 PMC7267315

[5] D. E. Gordon, G. M. Jang, M. Bouhaddou, J. Xu, K. Obernier, K. M. White, M. J. O’Meara, V. V. Rezelj, J. Z. Guo, D. L. Swaney, T. A. Tummino, R. Hüttenhain, R. M. Kaake, A. L. Richards, B. Tutuncuoglu, H. Foussard, J. Batra, K. Haas, M. Modak, M. Kim, P. Haas, B. J. Polacco, H. Braberg, J. M. Fabius, M. Eckhardt, M. Soucheray, M. J. Bennett, M. Cakir, M. J. McGregor, Q. Li, B. Meyer, F. Roesch, T. Vallet, A. Mac Kain, L. Miorin, E. Moreno, Z. Z. C. Naing, Y. Zhou, S. Peng, Y. Shi, Z. Zhang, W. Shen, I. T. Kirby, J. E. Melnyk, J. S. Chorba, K. Lou, S. A. Dai, I. Barrio-Hernandez, D. Memon, C. Hernandez-Armenta, J. Lyu, C. J. P. Mathy, T. Perica, K. B. Pilla, S. J. Ganesan, D. J. Saltzberg, R. Rakesh, X. Liu, S. B. Rosenthal, L. Calviello, S. Venkataramanan, J. Liboy-Lugo, Y. Lin, X.-P. Huang, Y. Liu, S. A. Wankowicz, M. Bohn, M. Safari, F. S. Ugur, C. Koh, N. S. Savar, Q. D. Tran, D. Shengjuler, S. J. Fletcher, M. C. O’Neal, Y. Cai, J. C. J. Chang, D. J. Broadhurst, S. Klippsten, P. P. Sharp, N. A. Wenzell, D. Kuzuoglu-Ozturk, H.-Y. Wang, R. Trenker, J. M. Young, D. A. Cavero, J. Hiatt, T. L. Roth, U. Rathore, A. Subramanian, J. Noack, M. Hubert, R. M. Stroud, A. D. Frankel, O. S. Rosenberg, K. A. Verba, D. A. Agard, M. Ott, M. Emerman, N. Jura, M. von Zastrow, E. Verdin, A. Ashworth, O. Schwartz, C. d’Enfert, S. Mukherjee, M. Jacobson, H. S. Malik, D. G. Fujimori, T. Ideker, C. S. Craik, S. N. Floor, J. S. Fraser, J. D. Gross, A. Sali, B. L. Roth, D. Ruggero, J. Taunton, T. Kortemme, P. Beltrao, M. Vignuzzi, A. García-Sastre, K. M. Shokat, B. K. Shoichet, N. J. Krogan, A SARS-CoV-2 protein interaction map reveals targets for drug repurposing. Nature 583, 459–468 (2020). 10.1038/s41586-020-2286-9 32353859 PMC7431030

[6] G. Teo, G. Liu, J. Zhang, A. I. Nesvizhskii, A.-C. Gingras, H. Choi, SAINTexpress: Improvements and additional features in Significance Analysis of INTeractome software. J. Proteomics 100, 37–43 (2014). 10.1016/j.jprot.2013.10.023 24513533 PMC4102138

[7] S. Jäger, P. Cimermancic, N. Gulbahce, J. R. Johnson, K. E. McGovern, S. C. Clarke, M. Shales, G. Mercenne, L. Pache, K. Li, H. Hernandez, G. M. Jang, S. L. Roth, E. Akiva, J. Marlett, M. Stephens, I. D’Orso, J. Fernandes, M. Fahey, C. Mahon, A. J. O’Donoghue, A. Todorovic, J. H. Morris, D. A. Maltby, T. Alber, G. Cagney, F. D. Bushman, J. A. Young, S. K. Chanda, W. I. Sundquist, T. Kortemme, R. D. Hernandez, C. S. Craik, A. Burlingame, A. Sali, A. D. Frankel, N. J. Krogan, Global landscape of HIV-human protein complexes. Nature 481, 365–370 (2011). 10.1038/nature10719 22190034 PMC3310911

[8] J. C. Young, N. J. Hoogenraad, F. U. Hartl, Molecular chaperones Hsp90 and Hsp70 deliver preproteins to the mitochondrial import receptor Tom70. Cell 112, 41–50 (2003). 10.1016/S0092-8674(02)01250-3 12526792

[9] R. Lin, S. Paz, J. Hiscott, Tom70 imports antiviral immunity to the mitochondria. Cell Res. 20, 971–973 (2010). 10.1038/cr.2010.113 20680033

[10] B. Wei, Y. Cui, Y. Huang, H. Liu, L. Li, M. Li, K.-C. Ruan, Q. Zhou, C. Wang, Tom70 mediates Sendai virus-induced apoptosis on mitochondria. J. Virol. 89, 3804–3818 (2015). 10.1128/JVI.02959-14 25609812 PMC4403418

[11] A. M. Edmonson, D. K. Mayfield, V. Vervoort, B. R. DuPont, G. Argyropoulos, Characterization of a human import component of the mitochondrial outer membrane, TOMM70A. Cell Commun. Adhes. 9, 15–27 (2002). 10.1080/15419060212186 12200962

[12] M. J. Baker, A. E. Frazier, J. M. Gulbis, M. T. Ryan, Mitochondrial protein-import machinery: Correlating structure with function. Trends Cell Biol. 17, 456–464 (2007). 10.1016/j.tcb.2007.07.010 17825565

[13] J. Brix, K. Dietmeier, N. Pfanner, Differential recognition of preproteins by the purified cytosolic domains of the mitochondrial import receptors Tom20, Tom22, and Tom70. J. Biol. Chem. 272, 20730–20735 (1997). 10.1074/jbc.272.33.20730 9252394

[14] J. Brix, G. A. Ziegler, K. Dietmeier, J. Schneider-Mergener, G. E. Schulz, N. Pfanner, The mitochondrial import receptor Tom70: Identification of a 25 kDa core domain with a specific binding site for preproteins. J. Mol. Biol. 303, 479–488 (2000). 10.1006/jmbi.2000.4120 11054285

[15] R. D. Mills, J. Trewhella, T. W. Qiu, T. Welte, T. M. Ryan, T. Hanley, R. B. Knott, T. Lithgow, T. D. Mulhern, Domain organization of the monomeric form of the Tom70 mitochondrial import receptor. J. Mol. Biol. 388, 1043–1058 (2009). 10.1016/j.jmb.2009.03.070 19358854

[16] S. D. Weeks, S. De Graef, A. Munawar, X-ray Crystallographic Structure of Orf9b from SARS-CoV-2 (2020); 10.2210/pdb6z4u/pdb. 10.2210/pdb6z4u/pdb

[17] M. Bouhaddou, D. Memon, B. Meyer, K. M. White, V. V. Rezelj, M. Correa Marrero, B. J. Polacco, J. E. Melnyk, S. Ulferts, R. M. Kaake, J. Batra, A. L. Richards, E. Stevenson, D. E. Gordon, A. Rojc, K. Obernier, J. M. Fabius, M. Soucheray, L. Miorin, E. Moreno, C. Koh, Q. D. Tran, A. Hardy, R. Robinot, T. Vallet, B. E. Nilsson-Payant, C. Hernandez-Armenta, A. Dunham, S. Weigang, J. Knerr, M. Modak, D. Quintero, Y. Zhou, A. Dugourd, A. Valdeolivas, T. Patil, Q. Li, R. Hüttenhain, M. Cakir, M. Muralidharan, M. Kim, G. Jang, B. Tutuncuoglu, J. Hiatt, J. Z. Guo, J. Xu, S. Bouhaddou, C. J. P. Mathy, A. Gaulton, E. J. Manners, E. Félix, Y. Shi, M. Goff, J. K. Lim, T. McBride, M. C. O’Neal, Y. Cai, J. C. J. Chang, D. J. Broadhurst, S. Klippsten, E. De Wit, A. R. Leach, T. Kortemme, B. Shoichet, M. Ott, J. Saez-Rodriguez, B. R. tenOever, R. D. Mullins, E. R. Fischer, G. Kochs, R. Grosse, A. García-Sastre, M. Vignuzzi, J. R. Johnson, K. M. Shokat, D. L. Swaney, P. Beltrao, N. J. Krogan, The Global Phosphorylation Landscape of SARS-CoV-2 Infection. Cell 182, 685–712.e19 (2020). 10.1016/j.cell.2020.06.034 32645325 PMC7321036

[18] J. Li, X. Qian, J. Hu, B. Sha, Molecular chaperone Hsp70/Hsp90 prepares the mitochondrial outer membrane translocon receptor Tom71 for preprotein loading. J. Biol. Chem. 284, 23852–23859 (2009). 10.1074/jbc.M109.023986 19581297 PMC2749157

[19] X.-Y. Liu, B. Wei, H.-X. Shi, Y.-F. Shan, C. Wang, Tom70 mediates activation of interferon regulatory factor 3 on mitochondria. Cell Res. 20, 994–1011 (2010). 10.1038/cr.2010.103 20628368

[20] Y. Liu, C. Zhang, F. Huang, Y. Yang, F. Wang, J. Yuan, Z. Zhang, Y. Qin, X. Li, D. Zhao, S. Li, S. Tan, Z. Wang, J. Li, C. Shen, J. Li, L. Peng, W. Wu, M. Cao, L. Xing, Z. Xu, L. Chen, C. Zhou, W. J. Liu, L. Liu, C. Jiang, Elevated plasma levels of selective cytokines in COVID-19 patients reflect viral load and lung injury. Natl. Sci. Rev. 7, 1003–1011 (2020). 10.1093/nsr/nwaa037 PMC7107806 34676126

[21] C. Huang, Y. Wang, X. Li, L. Ren, J. Zhao, Y. Hu, L. Zhang, G. Fan, J. Xu, X. Gu, Z. Cheng, T. Yu, J. Xia, Y. Wei, W. Wu, X. Xie, W. Yin, H. Li, M. Liu, Y. Xiao, H. Gao, L. Guo, J. Xie, G. Wang, R. Jiang, Z. Gao, Q. Jin, J. Wang, B. Cao, Clinical features of patients infected with 2019 novel coronavirus in Wuhan, China. Lancet 395, 497–506 (2020). 10.1016/S0140-6736(20)30183-5 31986264 PMC7159299

[22] C. Qin, L. Zhou, Z. Hu, S. Zhang, S. Yang, Y. Tao, C. Xie, K. Ma, K. Shang, W. Wang, D.-S. Tian, Dysregulation of Immune Response in Patients With Coronavirus 2019 (COVID-19) in Wuhan, China. Clin. Infect. Dis. 71, 762–768 (2020). 10.1093/cid/ciaa248 32161940 PMC7108125

[23] G. Chen, D. Wu, W. Guo, Y. Cao, D. Huang, H. Wang, T. Wang, X. Zhang, H. Chen, H. Yu, X. Zhang, M. Zhang, S. Wu, J. Song, T. Chen, M. Han, S. Li, X. Luo, J. Zhao, Q. Ning, Clinical and immunological features of severe and moderate coronavirus disease 2019. J. Clin. Invest. 130, 2620–2629 (2020). 10.1172/JCI137244 32217835 PMC7190990

[24] M. Zaretsky, R. Etzyoni, J. Kaye, L. Sklair-Tavron, A. Aharoni, Directed Evolution of a Soluble Human IL-17A Receptor for the Inhibition of Psoriasis Plaque Formation in a Mouse Model. Chem. Biol. 20, 202–211 (2013). 10.1016/j.chembiol.2012.11.012 23438749

[25] M. Sohda, Y. Misumi, K. Tashiro, M. Yamazaki, T. Saku, K. Oda, Identification of a soluble isoform of human IL-17RA generated by alternative splicing. Cytokine 64, 642–645 (2013). 10.1016/j.cyto.2013.09.012 24084331

[26] J. Lokau, C. Garbers, Biological functions and therapeutic opportunities of soluble cytokine receptors. Cytokine Growth Factor Rev. 55, 94–108 (2020). 10.1016/j.cytogfr.2020.04.003 32386776

[27] M. Sammel, F. Peters, J. Lokau, F. Scharfenberg, L. Werny, S. Linder, C. Garbers, S. Rose-John, C. Becker-Pauly, Differences in Shedding of the Interleukin-11 Receptor by the Proteases ADAM9, ADAM10, ADAM17, Meprin α, Meprin β and MT1-MMP. Int. J. Mol. Sci. 20, 3677 (2019). 10.3390/ijms20153677 PMC6696353 31357561

[28] B. B. Sun, J. C. Maranville, J. E. Peters, D. Stacey, J. R. Staley, J. Blackshaw, S. Burgess, T. Jiang, E. Paige, P. Surendran, C. Oliver-Williams, M. A. Kamat, B. P. Prins, S. K. Wilcox, E. S. Zimmerman, A. Chi, N. Bansal, S. L. Spain, A. M. Wood, N. W. Morrell, J. R. Bradley, N. Janjic, D. J. Roberts, W. H. Ouwehand, J. A. Todd, N. Soranzo, K. Suhre, D. S. Paul, C. S. Fox, R. M. Plenge, J. Danesh, H. Runz, A. S. Butterworth, Genomic atlas of the human plasma proteome. Nature 558, 73–79 (2018). 10.1038/s41586-018-0175-2 29875488 PMC6697541

[29] Z. Zhu, Z. Zheng, F. Zhang, Y. Wu, M. Trzaskowski, R. Maier, M. R. Robinson, J. J. McGrath, P. M. Visscher, N. R. Wray, J. Yang, Causal associations between risk factors and common diseases inferred from GWAS summary data. Nat. Commun. 9, 224 (2018). 10.1038/s41467-017-02317-2 29335400 PMC5768719

[30] The COVID-19 Host Genetics Initiative, The COVID-19 Host Genetics Initiative, a global initiative to elucidate the role of host genetic factors in susceptibility and severity of the SARS-CoV-2 virus pandemic. Eur. J. Hum. Genet. 28, 715–718 (2020). 10.1038/s41431-020-0636-6 32404885 PMC7220587

[31] B. E. Young, S.-W. Fong, Y.-H. Chan, T.-M. Mak, L. W. Ang, D. E. Anderson, C. Y.-P. Lee, S. N. Amrun, B. Lee, Y. S. Goh, Y. C. F. Su, W. E. Wei, S. Kalimuddin, L. Y. A. Chai, S. Pada, S. Y. Tan, L. Sun, P. Parthasarathy, Y. Y. C. Chen, T. Barkham, R. T. P. Lin, S. Maurer-Stroh, Y.-S. Leo, L.-F. Wang, L. Renia, V. J. Lee, G. J. D. Smith, D. C. Lye, L. F. P. Ng, Effects of a major deletion in the SARS-CoV-2 genome on the severity of infection and the inflammatory response: An observational cohort study. Lancet 396, 603–611 (2020). 10.1016/S0140-6736(20)31757-8 32822564 PMC7434477

[32] C. Amici, A. Di Caro, A. Ciucci, L. Chiappa, C. Castilletti, V. Martella, N. Decaro, C. Buonavoglia, M. R. Capobianchi, M. G. Santoro, Indomethacin has a potent antiviral activity against SARS coronavirus. Antivir. Ther. 11, 1021–1030 (2006). 17302372

[33] P. R. Rosenbaum, D. B. Rubin, The central role of the propensity score in observational studies for causal effects. Biometrika 70, 41–55 (1983). 10.1093/biomet/70.1.41

[34] C. Abate, P. D. Mosier, F. Berardi, R. A. Glennon, A structure-affinity and comparative molecular field analysis of sigma-2 (σ2) receptor ligands. Cent. Nerv. Syst. Agents Med. Chem. 9, 246–257 (2009). 10.2174/1871524910909030246 20021358

[35] R. A. Glennon, Sigma receptor ligands and the use thereof, U.S. Patent 6,057,371 (2000); https://patentimages.storage.googleapis.com/dc/36/68/73f4ccdac4c973/US6057371.pdf.

[36] R. R. Matsumoto, B. Pouw, Correlation between neuroleptic binding to σ_1_ and σ_2_ receptors and acute dystonic reactions. Eur. J. Pharmacol. 401, 155–160 (2000). 10.1016/S0014-2999(00)00430-1 10924920

[37] M. Dold, M. T. Samara, C. Li, M. Tardy, S. Leucht, Haloperidol versus first-generation antipsychotics for the treatment of schizophrenia and other psychotic disorders. Cochrane Database Syst. Rev. 1, CD009831 (2015). 10.1002/14651858.CD009831.pub2 25592299 PMC10787950

[38] F. F. Moebius, R. J. Reiter, K. Bermoser, H. Glossmann, S. Y. Cho, Y. K. Paik, Pharmacological analysis of sterol delta8-delta7 isomerase proteins with [3H]ifenprodil. Mol. Pharmacol. 54, 591–598 (1998). 10.1124/mol.54.3.591 9730919

[39] E. Gregori-Puigjané, V. Setola, J. Hert, B. A. Crews, J. J. Irwin, E. Lounkine, L. Marnett, B. L. Roth, B. K. Shoichet, Identifying mechanism-of-action targets for drugs and probes. Proc. Natl. Acad. Sci. U.S.A. 109, 11178–11183 (2012). 10.1073/pnas.1204524109 22711801 PMC3396511

[40] Z. Hubler, D. Allimuthu, I. Bederman, M. S. Elitt, M. Madhavan, K. C. Allan, H. E. Shick, E. Garrison, M. T Karl, D. C. Factor, Z. S. Nevin, J. L. Sax, M. A. Thompson, Y. Fedorov, J. Jin, W. K. Wilson, M. Giera, F. Bracher, R. H. Miller, P. J. Tesar, D. J. Adams, Accumulation of 8,9-unsaturated sterols drives oligodendrocyte formation and remyelination. Nature 560, 372–376 (2018). 10.1038/s41586-018-0360-3 30046109 PMC6423962

[41] F. F. Moebius, R. J. Reiter, M. Hanner, H. Glossmann, High affinity of sigma 1-binding sites for sterol isomerization inhibitors: Evidence for a pharmacological relationship with the yeast sterol C8-C7 isomerase. Br. J. Pharmacol. 121, 1–6 (1997). 10.1038/sj.bjp.0701079 9146879 PMC1564641

[42] H.-W. Jiang, H.-N. Zhang, Q.-F. Meng, J. Xie, Y. Li, H. Chen, Y.-X. Zheng, X.-N. Wang, H. Qi, J. Zhang, P.-H. Wang, Z.-G. Han, S.-C. Tao, SARS-CoV-2 Orf9b suppresses type I interferon responses by targeting TOM70. Cell. Mol. Immunol. 17, 998–1000 (2020). 10.1038/s41423-020-0514-8 32728199 PMC7387808

[43] J. J. Almagro Armenteros, C. K. Sønderby, S. K. Sønderby, H. Nielsen, O. Winther, DeepLoc: Prediction of protein subcellular localization using deep learning. Bioinformatics 33, 3387–3395 (2017). 10.1093/bioinformatics/btx431 29036616

[44] C. Chiva, R. Olivella, E. Borràs, G. Espadas, O. Pastor, A. Solé, E. Sabidó, QCloud: A cloud-based quality control system for mass spectrometry-based proteomics laboratories. PLOS ONE 13, e0189209 (2018). 10.1371/journal.pone.0189209 29324744 PMC5764250

[45] J. Cox, M. Mann, MaxQuant enables high peptide identification rates, individualized p.p.b.-range mass accuracies and proteome-wide protein quantification. Nat. Biotechnol. 26, 1367–1372 (2008). 10.1038/nbt.1511 19029910

[46] M. Giurgiu, J. Reinhard, B. Brauner, I. Dunger-Kaltenbach, G. Fobo, G. Frishman, C. Montrone, A. Ruepp, CORUM: The comprehensive resource of mammalian protein complexes-2019. Nucleic Acids Res. 47, D559–D563 (2019). 10.1093/nar/gky973 30357367 PMC6323970

[47] E. L. Huttlin, L. Ting, R. J. Bruckner, F. Gebreab, M. P. Gygi, J. Szpyt, S. Tam, G. Zarraga, G. Colby, K. Baltier, R. Dong, V. Guarani, L. P. Vaites, A. Ordureau, R. Rad, B. K. Erickson, M. Wühr, J. Chick, B. Zhai, D. Kolippakkam, J. Mintseris, R. A. Obar, T. Harris, S. Artavanis-Tsakonas, M. E. Sowa, P. De Camilli, J. A. Paulo, J. W. Harper, S. P. Gygi, The BioPlex Network: A Systematic Exploration of the Human Interactome. Cell 162, 425–440 (2015). 10.1016/j.cell.2015.06.043 26186194 PMC4617211

[48] G. Yu, L.-G. Wang, Y. Han, Q.-Y. He, clusterProfiler: An R package for comparing biological themes among gene clusters. OMICS 16, 284–287 (2012). 10.1089/omi.2011.0118 22455463 PMC3339379

[49] M. Remmert, A. Biegert, A. Hauser, J. Söding, HHblits: Lightning-fast iterative protein sequence searching by HMM-HMM alignment. Nat. Methods 9, 173–175 (2011). 10.1038/nmeth.1818 22198341

[50] J. Yang, I. Anishchenko, H. Park, Z. Peng, S. Ovchinnikov, D. Baker, Improved protein structure prediction using predicted interresidue orientations. Proc. Natl. Acad. Sci. U.S.A. 117, 1496–1503 (2020). 10.1073/pnas.1914677117 31896580 PMC6983395

[51] Y. Zhai, F. Sun, X. Li, H. Pang, X. Xu, M. Bartlam, Z. Rao, Insights into SARS-CoV transcription and replication from the structure of the nsp7-nsp8 hexadecamer. Nat. Struct. Mol. Biol. 12, 980–986 (2005). 10.1038/nsmb999 16228002 PMC7096913

[52] A. Waterhouse, M. Bertoni, S. Bienert, G. Studer, G. Tauriello, R. Gumienny, F. T. Heer, T. A. P. de Beer, C. Rempfer, L. Bordoli, R. Lepore, T. Schwede, SWISS-MODEL: Homology modelling of protein structures and complexes. Nucleic Acids Res. 46, W296–W303 (2018). 10.1093/nar/gky427 29788355 PMC6030848

[53] J. Durairaj, M. Akdel, D. de Ridder, A. D. J. van Dijk, Geometricus Represents Protein Structures as Shape-mers Derived from Moment Invariants. bioRxiv 2020.09.07.285569 [Preprint]. 8 September 2020. . 10.1101/2020.09.07.285569 33381814

[54] M. Akdel, J. Durairaj, D. de Ridder, A. D. J. van Dijk, Caretta - A multiple protein structure alignment and feature extraction suite. Comput. Struct. Biotechnol. J. 18, 981–992 (2020). 10.1016/j.csbj.2020.03.011 32368333 PMC7186369

[55] P. Shannon, A. Markiel, O. Ozier, N. S. Baliga, J. T. Wang, D. Ramage, N. Amin, B. Schwikowski, T. Ideker, Cytoscape: A software environment for integrated models of biomolecular interaction networks. Genome Res. 13, 2498–2504 (2003). 10.1101/gr.1239303 14597658 PMC403769

[56] R. T. Pillich, J. Chen, V. Rynkov, D. Welker, D. Pratt, NDEx: A Community Resource for Sharing and Publishing of Biological Networks. Methods Mol. Biol. 1558, 271–301 (2017). 10.1007/978-1-4939-6783-4_13 28150243

[57] D. K. W. Chu, Y. Pan, S. M. S. Cheng, K. P. Y. Hui, P. Krishnan, Y. Liu, D. Y. M. Ng, C. K. C. Wan, P. Yang, Q. Wang, M. Peiris, L. L. M. Poon, Molecular Diagnosis of a Novel Coronavirus (2019-nCoV) Causing an Outbreak of Pneumonia. Clin. Chem. 66, 549–555 (2020). 10.1093/clinchem/hvaa029 32031583 PMC7108203

[58] K. J. Livak, T. D. Schmittgen, Analysis of relative gene expression data using real-time quantitative PCR and the 2(-Delta Delta C(T)) Method. Methods 25, 402–408 (2001). 10.1006/meth.2001.1262 11846609

[59] R. Stoner, T. Maures, D. Conant, Methods and systems for guide RNA design and use, U.S. Patent 2019/0382797 A1 (2019); https://patentimages.storage.googleapis.com/95/c7/43/3d48387ce0f116/US20190382797A1.pdf.

[60] T. Hsiau, D. Conant, N. Rossi, T. Maures, K. Waite, J. Yang, S. Joshi, R. Kelso, K. Holden, B. L. Enzmann, R. Stoner, Inference of CRISPR Edits from Sanger Trace Data. bioRxiv 251082 [Preprint]. 10 August 2018. . 10.1101/251082 35119294

[61] A. S. Jureka, J. A. Silvas, C. F. Basler, Propagation, Inactivation, and Safety Testing of SARS-CoV-2. Viruses 12, 622 (2020). 10.3390/v12060622 32517266 PMC7354523

[62] A. C. Y. Fan, M. K. Bhangoo, J. C. Young, Hsp90 functions in the targeting and outer membrane translocation steps of Tom70-mediated mitochondrial import. J. Biol. Chem. 281, 33313–33324 (2006). 10.1074/jbc.M605250200 16968702

[63] S. Backes, S. Hess, F. Boos, M. W. Woellhaf, S. Gödel, M. Jung, T. Mühlhaus, J. M. Herrmann, Tom70 enhances mitochondrial preprotein import efficiency by binding to internal targeting sequences. J. Cell Biol. 217, 1369–1382 (2018). 10.1083/jcb.201708044 29382700 PMC5881500

[64] J. J. Almagro Armenteros, M. Salvatore, O. Emanuelsson, O. Winther, G. von Heijne, A. Elofsson, H. Nielsen, Detecting sequence signals in targeting peptides using deep learning. Life Sci. Alliance 2, e201900429 (2019). 10.26508/lsa.201900429 31570514 PMC6769257

[65] Y. Fukasawa, J. Tsuji, S.-C. Fu, K. Tomii, P. Horton, K. Imai, MitoFates: Improved prediction of mitochondrial targeting sequences and their cleavage sites. Mol. Cell. Proteomics 14, 1113–1126 (2015). 10.1074/mcp.M114.043083 25670805 PMC4390256

[66] A. Drozdetskiy, C. Cole, J. Procter, G. J. Barton, JPred4: A protein secondary structure prediction server. Nucleic Acids Res. 43, W389–W394 (2015). 10.1093/nar/gkv332 25883141 PMC4489285

[67] D. N. Mastronarde, Automated electron microscope tomography using robust prediction of specimen movements. J. Struct. Biol. 152, 36–51 (2005). 10.1016/j.jsb.2005.07.007 16182563

[68] S. Q. Zheng, E. Palovcak, J.-P. Armache, K. A. Verba, Y. Cheng, D. A. Agard, MotionCor2: Anisotropic correction of beam-induced motion for improved cryo-electron microscopy. Nat. Methods 14, 331–332 (2017). 10.1038/nmeth.4193 28250466 PMC5494038

[69] S. F. Altschul, T. L. Madden, A. A. Schäffer, J. Zhang, Z. Zhang, W. Miller, D. J. Lipman, Gapped BLAST and PSI-BLAST: A new generation of protein database search programs. Nucleic Acids Res. 25, 3389–3402 (1997). 10.1093/nar/25.17.3389 9254694 PMC146917

[70] P. Emsley, K. Cowtan, Coot: Model-building tools for molecular graphics. Acta Cryst. D60, 2126–2132 (2004). 10.1107/S0907444904019158 15572765

[71] R. Y.-R. Wang, Y. Song, B. A. Barad, Y. Cheng, J. S. Fraser, F. DiMaio, Automated structure refinement of macromolecular assemblies from cryo-EM maps using Rosetta. eLife 5, e17219 (2016). 10.7554/eLife.17219 27669148 PMC5115868

[72] R. T. Kidmose, J. Juhl, P. Nissen, T. Boesen, J. L. Karlsen, B. P. Pedersen, *Namdinator* - automatic molecular dynamics flexible fitting of structural models into cryo-EM and crystallography experimental maps. IUCrJ 6, 526–531 (2019). 10.1107/S2052252519007619 31316797 PMC6608625

[73] T. I. Croll, ISOLDE: A physically realistic environment for model building into low-resolution electron-density maps. Acta Cryst. D74, 519–530 (2018). 10.1107/S2059798318002425 29872003 PMC6096486

[74] T. D. Goddard, C. C. Huang, E. C. Meng, E. F. Pettersen, G. S. Couch, J. H. Morris, T. E. Ferrin, UCSF ChimeraX: Meeting modern challenges in visualization and analysis. Protein Sci. 27, 14–25 (2018). 10.1002/pro.3235 28710774 PMC5734306

[75] P. V. Afonine, B. P. Klaholz, N. W. Moriarty, B. K. Poon, O. V. Sobolev, T. C. Terwilliger, P. D. Adams, A. Urzhumtsev, New tools for the analysis and validation of cryo-EM maps and atomic models. Acta Cryst. D74, 814–840 (2018). 10.1107/S2059798318009324 30198894 PMC6130467

[76] E. Krissinel, K. Henrick, Inference of macromolecular assemblies from crystalline state. J. Mol. Biol. 372, 774–797 (2007). 10.1016/j.jmb.2007.05.022 17681537

[77] A. N. Honko, N. Storm, D. J. Bean, J. H. Vasquez, S. N. Downs, A. Griffiths, Rapid Quantification and Neutralization Assays for Novel Coronavirus SARS-CoV-2 Using Avicel RC-591 Semi-Solid Overlay. *Preprints* 2020050264 [Preprint]. 16 May 2020. www.preprints.org/manuscript/202005.0264/v1.

[78] A. Šali, T. L. Blundell, Comparative protein modelling by satisfaction of spatial restraints. J. Mol. Biol. 234, 779–815 (1993). 10.1006/jmbi.1993.1626 8254673

[79] T. Yamada, J. Komoto, K. Watanabe, Y. Ohmiya, F. Takusagawa, Crystal structure and possible catalytic mechanism of microsomal prostaglandin E synthase type 2 (mPGES-2). J. Mol. Biol. 348, 1163–1176 (2005). 10.1016/j.jmb.2005.03.035 15854652

[80] W. Yin, C. Mao, X. Luan, D.-D. Shen, Q. Shen, H. Su, X. Wang, F. Zhou, W. Zhao, M. Gao, S. Chang, Y.-C. Xie, G. Tian, H.-W. Jiang, S.-C. Tao, J. Shen, Y. Jiang, H. Jiang, Y. Xu, S. Zhang, Y. Zhang, H. E. Xu, Structural basis for inhibition of the RNA-dependent RNA polymerase from SARS-CoV-2 by remdesivir. Science 368, 1499–1504 (2020). 10.1126/science.abc1560 32358203 PMC7199908

[81] D. Kozakov, D. R. Hall, B. Xia, K. A. Porter, D. Padhorny, C. Yueh, D. Beglov, S. Vajda, The ClusPro web server for protein-protein docking. Nat. Protoc. 12, 255–278 (2017). 10.1038/nprot.2016.169 28079879 PMC5540229

[82] B. G. Pierce, K. Wiehe, H. Hwang, B.-H. Kim, T. Vreven, Z. Weng, ZDOCK server: Interactive docking prediction of protein-protein complexes and symmetric multimers. Bioinformatics 30, 1771–1773 (2014). 10.1093/bioinformatics/btu097 24532726 PMC4058926

[83] Y. Yan, H. Tao, J. He, S.-Y. Huang, The HDOCK server for integrated protein-protein docking. Nat. Protoc. 15, 1829–1852 (2020). 10.1038/s41596-020-0312-x 32269383

[84] A. Tovchigrechko, I. A. Vakser, GRAMM-X public web server for protein-protein docking. Nucleic Acids Res. 34, W310–W314 (2006). 10.1093/nar/gkl206 16845016 PMC1538913

[85] M. Torchala, I. H. Moal, R. A. G. Chaleil, J. Fernandez-Recio, P. A. Bates, SwarmDock: A server for flexible protein-protein docking. Bioinformatics 29, 807–809 (2013). 10.1093/bioinformatics/btt038 23343604

[86] D. Schneidman-Duhovny, Y. Inbar, R. Nussinov, H. J. Wolfson, PatchDock and SymmDock: Servers for rigid and symmetric docking. Nucleic Acids Res. 33, W363–W367 (2005). 10.1093/nar/gki481 15980490 PMC1160241

[87] G. Q. Dong, H. Fan, D. Schneidman-Duhovny, B. Webb, A. Sali, Optimized atomic statistical potentials: Assessment of protein interfaces and loops. Bioinformatics 29, 3158–3166 (2013). 10.1093/bioinformatics/btt560 24078704 PMC3842762

[88] J. Armstrong, G. Hickey, M. Diekhans, A. Deran, Q. Fang, D. Xie, S. Feng, J. Stiller, D. Genereux, J. Johnson, V. D. Marinescu, D. Haussler, J. Alföldi, K. Lindblad-Toh, E. Karlsson, E. D. Jarvis, G. Zhang, B. Paten, Progressive alignment with Cactus: A multiple-genome aligner for the thousand-genome era. bioRxiv 730531 [Preprint]. 15 October 2019. 10.1101/730531. 10.1101/730531

[89] B. Paten, D. Earl, N. Nguyen, M. Diekhans, D. Zerbino, D. Haussler, Cactus: Algorithms for genome multiple sequence alignment. Genome Res. 21, 1512–1528 (2011). 10.1101/gr.123356.111 21665927 PMC3166836

[90] M. D. Smith, J. O. Wertheim, S. Weaver, B. Murrell, K. Scheffler, S. L. Kosakovsky Pond, Less is more: An adaptive branch-site random effects model for efficient detection of episodic diversifying selection. Mol. Biol. Evol. 32, 1342–1353 (2015). 10.1093/molbev/msv022 25697341 PMC4408413

[91] S. L. K. Pond, S. D. W. Frost, S. V. Muse, HyPhy: Hypothesis testing using phylogenies. Bioinformatics 21, 676–679 (2005). 10.1093/bioinformatics/bti079 15509596

[92] K. S. Pollard, M. J. Hubisz, K. R. Rosenbloom, A. Siepel, Detection of nonneutral substitution rates on mammalian phylogenies. Genome Res. 20, 110–121 (2010). 10.1101/gr.097857.109 19858363 PMC2798823

[93] M. J. Hubisz, K. S. Pollard, A. Siepel, PHAST and RPHAST: Phylogenetic analysis with space/time models. Brief. Bioinform. 12, 41–51 (2011). 10.1093/bib/bbq072 21278375 PMC3030812

[94] R. Ramani, K. Krumholz, Y.-F. Huang, A. Siepel, PhastWeb: A web interface for evolutionary conservation scoring of multiple sequence alignments using phastCons and phyloP. Bioinformatics 35, 2320–2322 (2019). 10.1093/bioinformatics/bty966 30481262 PMC6596881

[95] W. A. Ray, Evaluating medication effects outside of clinical trials: New-user designs. Am. J. Epidemiol. 158, 915–920 (2003). 10.1093/aje/kwg231 14585769

[96] S. Schneeweiss, A basic study design for expedited safety signal evaluation based on electronic healthcare data. Pharmacoepidemiol. Drug Saf. 19, 858–868 (2010). 10.1002/pds.1926 20681003 PMC2917262

[97] H. Quan, V. Sundararajan, P. Halfon, A. Fong, B. Burnand, J.-C. Luthi, L. D. Saunders, C. A. Beck, T. E. Feasby, W. A. Ghali, Coding algorithms for defining comorbidities in ICD-9-CM and ICD-10 administrative data. Med. Care 43, 1130–1139 (2005). 10.1097/01.mlr.0000182534.19832.83 16224307

[98] P. C. Austin, Balance diagnostics for comparing the distribution of baseline covariates between treatment groups in propensity-score matched samples. Stat. Med. 28, 3083–3107 (2009). 10.1002/sim.3697 19757444 PMC3472075

[99] World Health Organization (WHO), WHO R&D Blueprint, novel Coronavirus: COVID-19 Therapeutic Trial Synopsis (WHO, 2020); www.who.int/blueprint/priority-diseases/key-action/COVID-19_Treatment_Trial_Design_Master_Protocol_synopsis_Final_18022020.pdf.

[100] Y. Perez-Riverol, A. Csordas, J. Bai, M. Bernal-Llinares, S. Hewapathirana, D. J. Kundu, A. Inuganti, J. Griss, G. Mayer, M. Eisenacher, E. Pérez, J. Uszkoreit, J. Pfeuffer, T. Sachsenberg, S. Yilmaz, S. Tiwary, J. Cox, E. Audain, M. Walzer, A. F. Jarnuczak, T. Ternent, A. Brazma, J. A. Vizcaíno, The PRIDE database and related tools and resources in 2019: Improving support for quantification data. Nucleic Acids Res. 47, D442–D450 (2019). 10.1093/nar/gky1106 30395289 PMC6323896

[101] J. Li, X. Qian, J. Hu, B. Sha, Crystal structure of Tom71 complexed with Hsp82 C-terminal fragment (2009); 10.2210/pdb3fp2/pdb. 10.2210/pdb3fp2/pdb

